# Clustering of physical health multimorbidity in people with severe mental illness: An accumulated prevalence analysis of United Kingdom primary care data

**DOI:** 10.1371/journal.pmed.1003976

**Published:** 2022-04-20

**Authors:** Naomi Launders, Joseph F Hayes, Gabriele Price, David PJ Osborn

**Affiliations:** 1 Division of Psychiatry, UCL, London, United Kingdom; 2 Camden and Islington NHS Foundation Trust, London, United Kingdom; 3 Public Health England, Health Improvement Directorate, London, United Kingdom; University of Toronto, CANADA

## Abstract

**Background:**

People with severe mental illness (SMI) have higher rates of a range of physical health conditions, yet little is known regarding the clustering of physical health conditions in this population. We aimed to investigate the prevalence and clustering of chronic physical health conditions in people with SMI, compared to people without SMI.

**Methods and findings:**

We performed a cohort-nested accumulated prevalence study, using primary care data from the Clinical Practice Research Datalink (CPRD), which holds details of 39 million patients in the United Kingdom. We identified 68,783 adults with a primary care diagnosis of SMI (schizophrenia, bipolar disorder, or other psychoses) from 2000 to 2018, matched up to 1:4 to 274,684 patients without an SMI diagnosis, on age, sex, primary care practice, and year of registration at the practice. Patients had a median of 28.85 (IQR: 19.10 to 41.37) years of primary care observations. Patients with SMI had higher prevalence of smoking (27.65% versus 46.08%), obesity (24.91% versus 38.09%), alcohol misuse (3.66% versus 13.47%), and drug misuse (2.08% versus 12.84%) than comparators. We defined 24 physical health conditions derived from the Elixhauser and Charlson comorbidity indices and used logistic regression to investigate individual conditions and multimorbidity. We controlled for age, sex, region, and ethnicity and then additionally for health risk factors: smoking status, alcohol misuse, drug misuse, and body mass index (BMI). We defined multimorbidity clusters using multiple correspondence analysis (MCA) and K-means cluster analysis and described them based on the observed/expected ratio. Patients with SMI had higher odds of 19 of 24 conditions and a higher prevalence of multimorbidity (odds ratio (OR): 1.84; 95% confidence interval [CI]: 1.80 to 1.88, *p* < 0.001) compared to those without SMI, particularly in younger age groups (males aged 30 to 39: OR: 2.49; 95% CI: 2.27 to 2.73; *p* < 0.001; females aged 18 to 30: OR: 2.69; 95% CI: 2.36 to 3.07; *p* < 0.001). Adjusting for health risk factors reduced the OR of all conditions. We identified 7 multimorbidity clusters in those with SMI and 7 in those without SMI. A total of 4 clusters were common to those with and without SMI; while 1, heart disease, appeared as one cluster in those with SMI and 3 distinct clusters in comparators; and 2 small clusters were unique to the SMI cohort. Limitations to this study include missing data, which may have led to residual confounding, and an inability to investigate the temporal associations between SMI and physical health conditions.

**Conclusions:**

In this study, we observed that physical health conditions cluster similarly in people with and without SMI, although patients with SMI had higher burden of multimorbidity, particularly in younger age groups. While interventions aimed at the general population may also be appropriate for those with SMI, there is a need for interventions aimed at better management of younger-age multimorbidity, and preventative measures focusing on diseases of younger age, and reduction of health risk factors.

## Abstract 2

In an observational analysis of primary care data from the UK, Naomi Launders and colleagues study the prevalence and clustering of physical health conditions and multimorbidity in individuals with severe mental illnesses.

## Author summary

**Why was this study done?:**

- People with severe mental illness (SMI) such as schizophrenia, bipolar disorder, and other psychoses have more physical illnesses, such as diabetes or heart disease, than people without SMI.
- They are also more likely to have risk factors for poor health, such as smoking, obesity. or substance misuse.
- While we know that these patients have poorer physical health, we do not know whether the patterns of disease are the same as the general population.

**What did the researchers do and find?:**

- We used electronic medical records to investigate how common 24 physical illnesses are in people with SMI and used a mathematical model to compare them to people who did not have SMI.
- We then investigated how common it is to have multiple physical illnesses (multimorbidity) and which diseases are commonly found together in people with and without SMI.
- We found that people with SMI had more physical health conditions than people without SMI, particularly in younger age groups.
- We also found that physical conditions cluster similarly in people with and without SMI.

**What do these findings mean?:**

- We see similar clusters of physical health conditions in people with and without SMI, suggesting that focusing on diseases common in the general population will also reduce the burden of disease in those with SMI.
- In the general population, we think of multimorbidity occurring in older age, but the findings of our study suggest that there is a need to address younger-age multimorbidity and reduction of risk factors for poor physical health in people with SMI.

## Introduction

People with severe mental illness (SMI) are known to be at increased risk of a range of physical health conditions, at a younger age [1–3], and suffer worse outcomes related to these conditions [4]. Comorbidity has been well studied in people with SMI, and previous studies have found that people with SMI have a higher number of physical health conditions than the general population [5]. The challenges of the increased complexity of managing multiple physical health conditions [6–8] may disproportionally affect those with SMI, further increasing inequality in health outcomes [9,10] and increasing both secondary mental health and acute service use [11,12].

The concept of multimorbidity represents a shift from a single disease–centric approach to a more patient-centred view. Moving beyond disease pairs or counts of disease, and studying the way in which diseases and risk factors cluster within individuals, is crucial for improving patient outcomes through better diagnosis, treatment, and healthcare service provision [13,14]. There is currently not a common approach to the number or conditions studied, nor the methods used to describe multimorbidity [15,16]. The Academy of Medical Science has proposed a definition of multimorbidity that includes long-term physical health conditions, infectious diseases of long duration, and mental health conditions [17], while the National Institute for Health Care and Excellence (NICE) in England also includes risk factors for disease such as substance misuse [18].

While mental health diagnoses have been recognised as an important component of multimorbidity in the general population [6–8,15,19,20], there is a lack of evidence regarding the clustering of physical diseases in individuals with SMI or how profiles of physical health multimorbidity in this population compare to those without SMI.

Given the increased disease burden, poorer health outcomes, and higher mortality rate in people with SMI, it is important to characterise the disease profiles occurring in this population. We aimed to investigate the prevalence and clustering of chronic physical health conditions in people with SMI in a large national sample, compared to a matched comparator group without SMI, and investigate the impact of health risk factors in this population.

## Methods

### Population

We identified a cohort of patients from the Clinical Practice Research Datalink (CPRD) Gold and Aurum databases. These databases include primary care records for a subset of patients registered with primary care practices in the UK and have been shown to be broadly representative of the UK population [21,22]. At the time of this study, these databases contained deidentified electronic medical records for over 39 million patients. Ethical approval for this study was obtained from the Independent Scientific Advisory Committee of CPRD (protocol no. 18_288).

We included patients with a first diagnosis of SMI between 1 January 2000 and 31 December 2018 via medical codes for schizophrenia, bipolar disorder, or other nonaffective psychotic illnesses (S1 Code Lists [23]). Patients entered the cohort at the latest of registration with the primary care practice, age 18 or 1 January 2000 and exited the cohort at the earliest of end of registration, age 100, death or 31 December 2018. We excluded patients under the age of 18 at SMI diagnosis and those who had less than 1 year of active follow-up. Patients with SMI were matched to patients without SMI at a ratio of 1:1 to 1:4. Patients were matched strictly by sex, 5-year age band, primary care practice, and year of primary care practice registration and were required to be active in the database at the time of SMI diagnosis. Matching was performed by CPRD prior to receipt of the dataset.

### Study design

No prospective analysis plan for this study was documented; however, we identified the study aims, designed the study and planned the analyses and sensitivity analyses a priori. Following peer review, we performed an additional sensitivity analysis to investigate the impact of multiple imputation of ethnicity and changed the matching strategy from strict 1:4 matching, to allow cases to matched to comparators at a ratio of 1:1 up to 1:4. This study is reported as per the Strengthening the Reporting of Observational Studies in Epidemiology (STROBE) guideline (S1 Checklist).

### Outcomes

The primary outcomes were presence of physical health multimorbidity, defined as 2 or more of the studied conditions, and accumulated prevalence of 24 everdiagnosed chronic physical health conditions in people with SMI compared to the people without SMI. Diagnoses were as recorded in primary care.

We generated code lists for physical health conditions from code lists originally developed by Metcalf and colleagues [24] for calculating the Charlson and Elixhauser comorbidity indices. We made a number of modifications specific to considering physical health in people with SMI. We collapsed different severity levels of the same condition into one variable (e.g., uncomplicated diabetes and diabetes with complications were coded as diabetes), reducing the Charlson comorbidity index from 17 conditions to 14 and the Elixhauser index from 31 to 27. We also removed weight loss, obesity, alcohol misuse, and drug misuse from the Elixhauser index. We excluded psychoses and depression from the Elixhauser index and dementia from the Charlson comorbidity index as we were focusing on physical health. We then combined the 2 comorbidity indices into one list of 23 conditions (11 present in both lists, 10 unique to the Elixhauser index, and 2 unique to the Charlson comorbidity index). Finally, for chronic pulmonary disease, chronic obstructive pulmonary disease (COPD) and asthma were considered as separate conditions.

The final multimorbidity list (S1 Code Lists) consisted of 24 conditions: asthma, COPD, cardiac arrhythmia, congestive heart failure, myocardial infarction, cerebrovascular disease, neurological disorders (including epilepsy, multiple sclerosis, Parkinson disease, and seizures but excluding cerebrovascular disease and dementia), cancer, diabetes (type 1 or 2), hypothyroidism, liver disease, renal disease, peptic ulcers, rheumatic and collagen disease, paresis or paralysis, HIV/AIDS, hypertension, peripheral vascular disease, pulmonary circulation disorders, valvular disease, deficiency anaemia, blood loss anaemia, coagulopathy, and fluid or electrolyte disorders.

### Health risk factors for physical health conditions

We conceptualised alcohol misuse, drug misuse, smoking, and obesity as health risk factors for the development of physical health conditions. We defined alcohol and drug misuse using the code lists for the Elixhauser comorbidity index [24]. We categorised body mass index (BMI) as the heaviest ever recorded of obese (BMI ≥ 30), overweight (BMI 25 to 29.9), healthy weight (BMI 18.5 to 24.9), or underweight (BMI < 18.5), derived from specific medical code lists for obesity, recorded BMI, and BMI calculated from weight and height recording. We categorised smoking status as nonsmoker, ex-smoker, or current smoker using medical code lists, taking the most recent category and recording any nonsmokers with a historical code for smoking as ex-smokers.

### Covariates

We defined age as age at the end of follow-up based on year of birth. We considered age as a continuous variable and in 10-year age groups where results were stratified by age. Sex and ethnicity were as recorded in patient medical records and ethnicity was grouped as “Asian,” “Black,” “Mixed,” “White,” or “Other,” in line with UK 2011 Census Ethnic Group categories (https://www.ons.gov.uk/census/2011census/2011censusdata/2011censususerguide/variablesandclassifications). Where multiple ethnicities existed for an individual, we selected the most frequent, and where frequencies were equal, the most recent. Region was defined as the 9 English regions as listed by the Office for National Statistics and Scotland, Wales, and Northern Ireland and was based on primary care practice postcode.

### Missing data

As general practitioners are less likely to record values that are within the normal range [25,26], we coded patients with missing smoking or BMI data as nonsmoker or normal range BMI, respectively. We coded ethnicity, as recorded in primary care, as white ethnicity where this variable was missing [25]. This approach is in line with previous research using primary care data, which suggests that more than 93% of individuals without ethnicity recorded are from a white ethnic group [27]. We performed sensitivity analyses to assess the effect of coding missing ethnicity as missing rather than white and of using multiple imputation to estimate missing ethnicity.

### Analysis

We determined the prevalence of individual physical health conditions and pairs of conditions (e.g., hypertension and diabetes), stratified by SMI diagnosis, age, and sex. We used logistic regression to investigate the relative prevalence of each physical health condition, first controlling for age, sex, ethnicity, and region and then for these variables plus health risk factors: smoking status, BMI category, alcohol misuse, and drug misuse. We considered a 2-sided *p*-value of less than 0.05 to represent statistical significance, although due to the large number of observations, we examined effect size and confidence intervals (CIs) to interpret clinical significance.

We then undertook cluster analysis using the subset of patients with multimorbidity, stratified by presence or absence of an SMI diagnosis. We performed multiple correspondence analysis (MCA) to investigate the relationship between physical health conditions and to transform the discrete physical health conditions into continuous variables prior to cluster analysis. We then used the MCA dimensions in K-means cluster analysis to identify clusters of physical health conditions and assign individual patients to clusters. We determined the optimum number of clusters by visual inspection of both the Silhouettes and Calinski–Harabaz results. We described clusters using the variables with an observed/expected ratio of more than 1.2 or by variables for which more than 70% of patients with that variable were contained within the cluster. We then reran the MCA and cluster analysis with health risk factors included.

## Results

We identified 70,855 patients with a diagnosis of SMI. Of these, 273 patients were excluded as they did not meet the age criteria, 27 due to less than 1 year’s follow-up, 172 because SMI diagnosis was not within the study period, 1,571 because diagnosis was prior to age 18, and 29 because of missing practice details. Of the remaining 68,783 patients with SMI, 15,028 had a diagnosis of schizophrenia, 24,420 a diagnosis of bipolar disorder, and 29,335 a diagnosis of other psychoses. These patients were matched to 274,684 patients without SMI. A higher proportion of patients with SMI died during follow-up than comparators, and death occurred at a younger mean age (Table 1). A greater proportion of patients in the comparator group had missing information for ethnicity (43.3% versus 35.8%), smoking (7.0% versus 2.4%), and BMI (18.9% versus 9.8%) than in the SMI cohort.

**Table 1:** Characteristics of the SMI cohort and matched comparators, *n* = 349,072.

	No SMI	SMI			
		All SMI	Schizophrenia	Bipolar	Other
**Number of patients**	274,684	68,783	15,028	24,420	29,335
**Age at end of study (mean (SD))**	50.61 (19.59)	50.67 (19.06)	48.23 (17.30)	50.53 (17.28)	52.04 (21.10)
**Age at diagnosis (mean (SD))** ^**a**^	44.63 (18.77)	44.63 (18.80)	41.54 (17.06)	44.20 (16.46)	46.56 (21.11)
**Female (%)**	134,754 (49.05)	33,748 (49.06)	5,344 (35.56)	14,450 (59.17)	13,954 (47.57)
**Ethnicity (%)** ^**b**^					
Asian	13,864 (5.05)	3,251 (4.73)	1,063 (7.07)	783 (3.21)	1,405 (4.79)
Black	8,935 (3.25)	3,611 (5.25)	1,436 (9.56)	536 (2.19)	1,639 (5.59)
Mixed	2,118 (0.77)	840 (1.22)	238 (1.58)	251 (1.03)	351 (1.20)
Other	5,986 (2.18)	1,539 (2.24)	346 (2.30)	514 (2.10)	679 (2.31)
White	243,781 (88.75)	59,542 (86.56)	11,945 (79.48)	22,336 (91.47)	25,261 (86.11)
**BMI category (%)** ^**c**^					
Underweight	4,283 (1.56)	804 (1.17)	199 (1.32)	169 (0.69)	436 (1.49)
Healthy weight	127,237 (46.32)	22,622 (32.89)	4,871 (32.41)	6,790 (27.81)	10,961 (37.36)
Overweight	74,737 (27.21)	19,160 (27.86)	3,922 (26.10)	7,038 (28.82)	8,200 (27.95)
Obese	68,427 (24.91)	26,197 (38.09)	6,036 (40.17)	10,423 (42.68)	9,738 (33.20)
**Smoking category (%)** ^**d**^					
Current smoker	75,940 (27.65)	31,695 (46.08)	8,077 (53.75)	10,383 (42.52)	130,235 (45.12)
Ex-smoker	85,208 (31.02)	20,565 (29.90)	3,596 (23.93)	8,304 (34.00)	8,665 (29.54)
Never smoked	113,536 (41.33)	16,523 (24.02)	3,355 (22.32)	5,733 (23.48)	7,435 (25.35)
**Alcohol misuse (%)**	10,060 (3.66)	9,265 (13.47)	1,875 (12.48)	3,251 (13.31)	4,139 (14.11)
**Drug misuse (%)**	5,725 (2.08)	8,829 (12.84)	2,168 (14.43)	2,354 (9.64)	4,307 (14.68)
**Physical health conditions**					
No physical health conditions	139,312 (50.72)	27,029 (39.30)	6,766 (45.02)	8,928 (36.56)	11,335 (38.64)
One physical health condition	65,339 (23.79)	18,372 (26.71)	3,980 (26.48)	6,872 (28.14)	7,520 (25.63)
More than one physical health condition	70,033 (25.05)	23,382 (33.99)	4,282 (28.49)	8,620 (35.30)	10,480 (35.73)
**Deaths (%)**	19,536 (7.11)	7,581 (11.02)	1,617 (10.76)	2,158 (8.84)	3,806 (12.97)
**Age at death (mean, SD)**	79.03 (14.35)	71.57 (18.08)	66.06 (18.13)	70.14 (15.85)	74.72 (18.59)
**Years of observation (mean, SD)**	30.68 (16.90)	30.51 (16.62)	25.89 (16.12)	32.00 (16.13)	31.64 (16.86)

^a^For comparator population, this is the age of the individual at the time of diagnosis for the corresponding case.

^b^Missing ethnicity set to white (*n* = 143,642, 41.82%).

^c^Missing BMI set to normal (*n* = 58,551, 17.05%).

^d^Missing smoking set to nonsmoker (*n* = 20,967, 6.10%).

BMI, body mass index; SD, standard deviation; SMI, severe mental illness.

### Prevalence of chronic physical health conditions and multimorbidity

**Fig 1:**
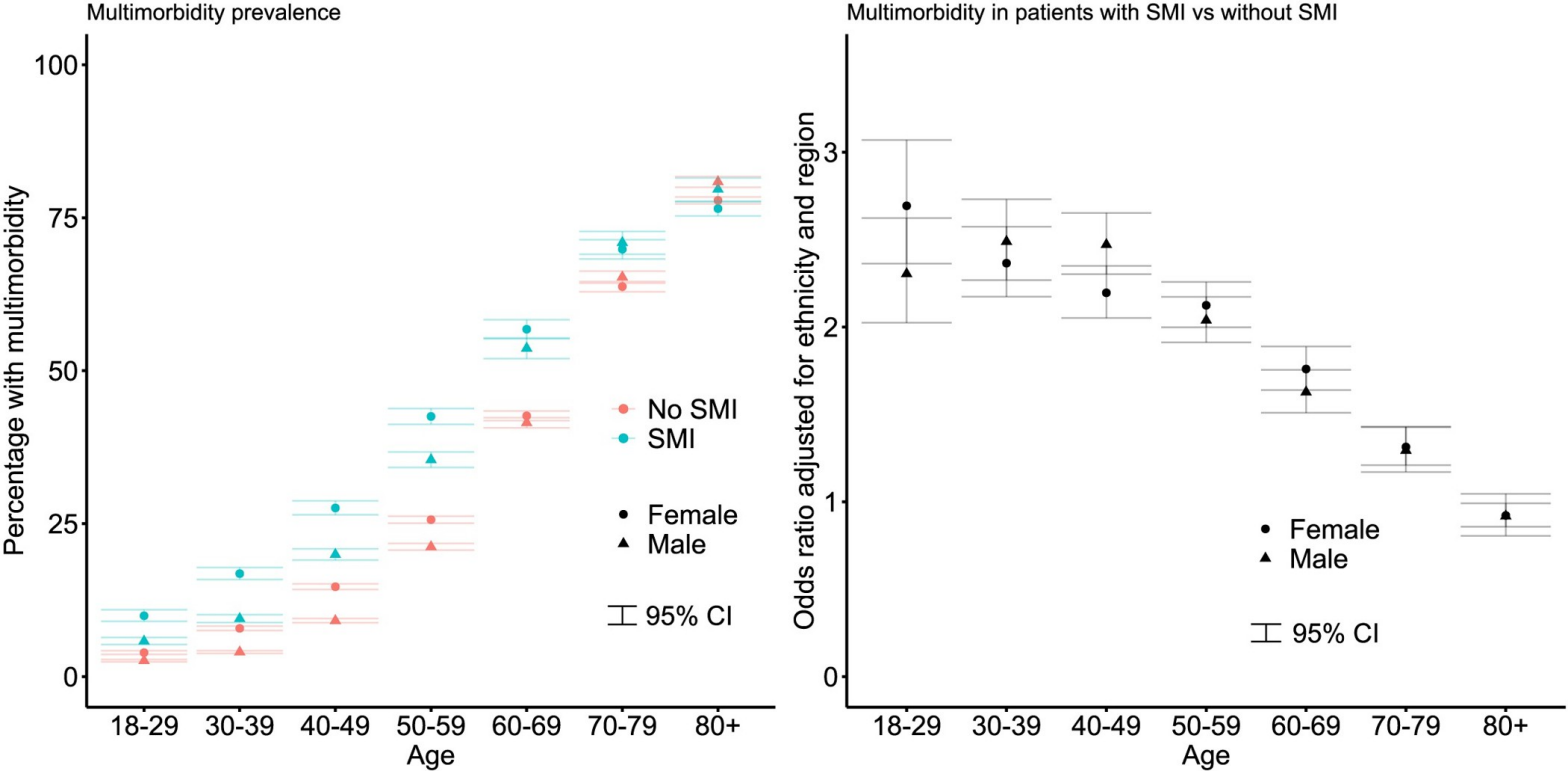
Prevalence of multimorbidity by SMI status, age group, and sex and OR of multimorbidity in those with SMI versus those without. CI, confidence interval; OR, odds ratio; SMI, severe mental illness.

There was a higher prevalence of at least 1 physical health condition and multimorbidity in the SMI cohort (Table 1). When controlling for age, sex, ethnicity, and region, those with SMI were at higher risk of multimorbidity (adjusted odds ratio [aOR]: 1.84; 95% CI: 1.80 to 1.88, *p* < 0.001). In both cohorts, multimorbidity was more common in females and in older age groups (Fig 1). The greatest difference in prevalence of multimorbidity between those with and without SMI was in patients aged 18 to 29 in females (aOR: 2.69; 95% CI: 2.36 to 3.07; *p* < 0.001) and 30 to 39 in males (aOR: 2.49; 95% CI: 2.27 to 2.73; *p* < 0.001; Fig 1). The difference got smaller with increasing age. In those age 80 and over, the prevalence of multimorbidity was similar in patients with and without SMI (Table 2, Fig 1). In patients aged 80 and over, patients with schizophrenia appeared at lower risk of multimorbidity compared to those without SMI (males: aOR: 0.39, 95% CI: 0.30 to 0.51, *p* < 0.001; females: aOR: 0.71, 95% CI: 0.60 to 0.84, *p* < 0.001; Table 2).

**Table 2:** Prevalence of multimorbidity stratified by age at end of follow-up and sex.

	Prevalence, *n* (%)					Unadjusted OR (95% CI)				Demographically aOR^a^ (95% CI)				Demographic and risk aOR^b^ (95% CI)			
	No SMI	SMI	Schiz^c^	Bipolar	Other	SMI	Schiz^c^	Bipolar	Other	SMI	Schiz^c^	Bipolar	Other	SMI	Schiz^c^	Bipolar	Other
Age at end of follow-up																	
MALE																	
18 to 29	701 (2.59)	372 (5.78)	91 (5.19)	75 (6.14)	206 (5.95)	2.30 (2.03 to 2.62) *p* < 0.001	2.05 (1.64 to 2.57) *p* < 0.001	2.46 (1.92 to 3.14) *p* < 0.001	2.38 (2.03 to 2.79) *p* < 0.001	2.30 (2.02 to 2.62) *p* < 0.001	2.05 (1.64 to 2.57) *p* < 0.001	2.48 (1.94 to 3.18) *p* < 0.001	2.37 (2.02 to 2.78) *p* < 0.001	1.76 (1.52 to 2.04) *p* < 0.001	1.48 (1.17 to 1.88) *p* = 0.001	1.88 (1.46 to 2.43) *p* < 0.001	1.87 (1.57 to 2.23) *p* < 0.001
30 to 39	1,315 (4.01)	774 (9.46)	250 (9.54)	165 (8.55)	359 (9.88)	2.50 (2.28 to 2.75) *p* < 0.001	2.53 (2.19 to 2.91) *p* < 0.001	2.24 (1.89 to 2.65) *p* < 0.001	2.63 (2.33 to 2.97) *p* < 0.001	2.49 (2.27 to 2.73) *p* < 0.001	2.50 (2.17 to 2.89) *p* < 0.001	2.27 (1.92 to 2.69) *p* < 0.001	2.59 (2.29 to 2.93) *p* < 0.001	1.47 (1.32 to 1.63) *p* < 0.001	1.41 (1.21 to 1.65) *p* < 0.001	1.36 (1.14 to 1.62) *p* < 0.001	1.58 (1.38 to 1.81) *p* < 0.001
40 to 49	2,532 (9.13)	1,453 (19.95)	435 (19.45)	415 (20.03)	603 (20.27)	2.48 (2.31 to 2.66) *p* < 0.001	2.40 (2.15 to 2.69) *p* < 0.001	2.49 (2.22 to 2.80) *p* < 0.001	2.53 (2.29 to 2.79) *p* < 0.001	2.47 (2.30 to 2.65) *p* < 0.001	2.33 (2.08 to 2.60) *p* < 0.001	2.56 (2.28 to 2.87) *p* < 0.001	2.52 (2.29 to 2.79) *p* < 0.001	1.47 (1.36 to 1.59) *p* < 0.001	1.40 (1.24 to 1.58) *p* < 0.001	1.49 (1.32 to 1.69) *p* < 0.001	1.52 (1.36 to 1.69) *p* < 0.001
50 to 59	4,609 (21.21)	1,996 (35.43)	522 (34.87)	686 (34.49)	788 (36.7)	2.04 (1.91 to 2.17) *p* < 0.001	1.99 (1.78 to 2.22) *p* < 0.001	1.96 (1.77 to 2.16) *p* < 0.001	2.15 (1.96 to 2.37) *p* < 0.001	2.04 (1.91 to 2.17) *p* < 0.001	1.91 (1.71 to 2.14) *p* < 0.001	2.02 (1.83 to 2.22) *p* < 0.001	2.15 (1.96 to 2.37) *p* < 0.001	1.38 (1.28 to 1.48) *p* < 0.001	1.32 (1.17 to 1.49) *p* < 0.001	1.34 (1.20 to 1.49) *p* < 0.001	1.47 (1.32 to 1.63) *p* < 0.001
60 to 69	5,719 (41.48)	1,854 (53.66)	401 (46.74)	746 (55.84)	707 (56.07)	1.63 (1.52 to 1.76) *p* < 0.001	1.24 (1.08 to 1.42) *p* = 0.002	1.78 (1.59 to 2.00) *p* < 0.001	1.80 (1.60 to 2.02) *p* < 0.001	1.63 (1.51 to 1.75) *p* < 0.001	1.20 (1.04 to 1.38) *p* = 0.01	1.82 (1.62 to 2.04) *p* < 0.001	1.78 (1.58 to 2.00) *p* < 0.001	1.28 (1.18 to 1.39) *p* < 0.001	0.96 (0.83 to 1.11) *p* = 0.59	1.36 (1.20 to 1.53) *p* < 0.001	1.47 (1.30 to 1.67) *p* < 0.001
70 to 79	5,995 (65.30)	1,639 (70.92)	293 (63.01)	670 (74.20)	676 (71.69)	1.30 (1.17 to 1.43) *p* < 0.001	0.91 (0.75 to 1.10) *p* = 0.31	1.53 (1.31 to 1.78) *p* < 0.001	1.35 (1.16 to 1.56) *p* < 0.001	1.29 (1.17 to 1.43) *p* < 0.001	0.87 (0.72 to 1.06) *p* = 0.16	1.57 (1.34 to 1.84) *p* < 0.001	1.33 (1.14 to 1.54) *p* < 0.001	1.24 (1.12 to 1.38) *p* < 0.001	0.87 (0.71 to 1.07) *p* = 0.19	1.42 (1.20 to 1.67) *p* < 0.001	1.33 (1.14 to 1.55) *p* < 0.001
80+	6,189 (80.87)	1,378 (79.65)	159 (63.1)	431 (83.04)	788 (82.17)	0.93 (0.81 to 1.05) *p* = 0.25	0.40 (0.31 to 0.53) *p* < 0.001	1.16 (0.91 to 1.47) *p* = 0.22	1.09 (0.92 to 1.30) *p* = 0.33	0.92 (0.81 to 1.05) *p* = 0.20	0.39 (0.30 to 0.51) *p* < 0.001	1.17 (0.93 to 1.49) *p* = 0.19	1.07 (0.90 to 1.28) *p* = 0.44	0.97 (0.84 to 1.11) *p* = 0.65	0.44 (0.33 to 0.58) *p* < 0.001	1.16 (0.90 to 1.49) *p* = 0.25	1.15 (0.96 to 1.39) *p* = 0.13
FEMALE																	
18 to 29	678 (3.92)	384 (9.94)	56 (10.92)	166 (9.04)	162 (10.70)	2.71 (2.38 to 3.08) *p* < 0.001	3.00 (2.25 to 4.01) *p* < 0.001	2.44 (2.04 to 2.91) *p* < 0.001	2.94 (2.45 to 3.52) *p* < 0.001	2.69 (2.36 to 3.07) *p* < 0.001	2.91 (2.18 to 3.89) *p* < 0.001	2.48 (2.07 to 2.96) *p* < 0.001	2.88 (2.40 to 3.45) *p* < 0.001	2.17 (1.89 to 2.50) *p* < 0.001	2.28 (1.69 to 3.08) *p* < 0.001	1.96 (1.63 to 2.36) *p* < 0.001	2.41 (2.00 to 2.91) *p* < 0.001
30 to 39	1,805 (7.89)	969 (16.83)	153 (17.13)	453 (15.66)	363 (18.41)	2.36 (2.17 to 2.57) *p* < 0.001	2.42 (2.02 to 2.89) *p* < 0.001	2.17 (1.94 to 2.42) *p* < 0.001	2.64 (2.33 to 2.98) *p* < 0.001	2.37 (2.17 to 2.57) *p* < 0.001	2.35 (1.96 to 2.82) *p* < 0.001	2.21 (1.98 to 2.47) *p* < 0.001	2.60 (2.29 to 2.94) *p* < 0.001	1.71 (1.56 to 1.87) *p* < 0.001	1.65 (1.36 to 2.00) *p* < 0.001	1.54 (1.37 to 1.74) *p* < 0.001	2.01 (1.76 to 2.29) *p* < 0.001
40 to 49	3,336 (14.69)	1,662 (27.56)	273 (27.08)	847 (28.39)	542 (26.58)	2.21 (2.07 to 2.36) *p* < 0.001	2.16 (1.87 to 2.49) *p* < 0.001	2.30 (2.11 to 2.51) *p* < 0.001	2.10 (1.89 to 2.34) *p* < 0.001	2.20 (2.05 to 2.35) *p* < 0.001	2.00 (1.73 to 2.32) *p* < 0.001	2.38 (2.18 to 2.60) *p* < 0.001	2.04 (1.83 to 2.27) *p* < 0.001	1.60 (1.49 to 1.72) *p* < 0.001	1.43 (1.23 to 1.66) *p* < 0.001	1.71 (1.56 to 1.88) *p* < 0.001	1.53 (1.37 to 1.71) *p* < 0.001
50 to 59	5,645 (25.64)	2,425 (42.52)	410 (43.25)	1,163 (43.28)	852 (41.20)	2.14 (2.02 to 2.28) *p* < 0.001	2.21 (1.94 to 2.52) *p* < 0.001	2.21 (2.04 to 2.40) *p* < 0.001	2.03 (1.85 to 2.23) *p* < 0.001	2.12 (2.00 to 2.26) *p* < 0.001	2.02 (1.77 to 2.31) *p* < 0.001	2.29 (2.11 to 2.49) *p* < 0.001	1.96 (1.79 to 2.16) *p* < 0.001	1.61 (1.51 to 1.72) *p* < 0.001	1.51 (1.32 to 1.74) *p* < 0.001	1.68 (1.54 to 1.83) *p* < 0.001	1.58 (1.43 to 1.74) *p* < 0.001
60 to 69	6,6612 (42.62)	2,263 (56.77)	378 (52.50)	1,009 (59.28)	876 (56.01)	1.77 (1.65 to 1.90) *p* < 0.001	1.49 (1.28 to 1.73) *p* < 0.001	1.96 (1.77 to 2.17) *p* < 0.001	1.71 (1.54 to 1.90) *p* < 0.001	1.76 (1.64 to 1.89) *p* < 0.001	1.41 (1.21 to 1.64) *p* < 0.001	2.02 (1.82 to 2.24) *p* < 0.001	1.68 (1.51 to 1.86) *p* < 0.001	1.46 (1.35 to 1.57) *p* < 0.001	1.13 (0.96 to 1.32) *p* = 0.15	1.61 (1.44 to 1.79) *p* < 0.001	1.47 (1.32 to 1.64) *p* < 0.001
70 to 79	8,187 (63.75)	2,308 (69.85)	378 (63.85)	937 (73.95)	993 (68.72)	1.32 (1.21 to 1.43) *p* < 0.001	1.00 (0.85 to 1.19) *p* = 0.96	1.61 (1.42 to 1.84) *p* < 0.001	1.25 (1.11 to 1.40) *p* < 0.001	1.31 (1.21 to 1.43) *p* < 0.001	0.97 (0.82 to 1.16) *p* = 0.75	1.64 (1.44 to 1.87) *p* < 0.001	1.24 (1.10 to 1.39) *p* < 0.001	1.26 (1.16 to 1.38) *p* < 0.001	0.92 (0.76 to 1.10) *p* = 0.35	1.50 (1.31 to 1.72) *p* < 0.001	1.25 (1.10 to 1.41) *p* < 0.001
80+	16,710 (77.84)	3,905 (76.49)	483 (72.09)	857 (79.13)	2,565 (76.52)	0.93 (0.86 to 1.00) *p* = 0.04	0.74 (0.62 to 0.87) *p* < 0.001	1.08 (0.93 to 1.25) *p* = 0.32	0.93 (0.85 to 1.01) *p* = 0.09	0.92 (0.86 to 0.99) *p* = 0.03	0.71 (0.60 to 0.84) *p* < 0.001	1.10 (0.95 to 1.280) *p* = 0.20	0.92 (0.84 to 1.00) *p* = 0.06	0.94 (0.88 to 1.02) *p* = 0.14	0.71 (0.59 to 0.85) *p* < 0.001	1.02 (0.87 to 1.19) *p* = 0.80	0.98 (0.90 to 1.07) *p* = 0.65

^a^Demographic adjusted: adjusted for ethnicity and region.

^b^Demographic and risk adjusted: adjusted for age, sex, ethnicity, and region and, additionally, for smoking status, BMI category, alcohol misuse, and drug misuse.

^c^Schizophrenia.

aOR, adjusted odds ratio; BMI, body mass index; CI, confidence interval; OR, odds ratio; SMI, severe mental illness.

Additionally, controlling for smoking status, BMI category, alcohol misuse, and drug misuse reduced the OR for multimorbidity in patients with SMI, although it was still elevated compared to comparators (aOR: 1.40; 95% CI: 1.37 to 1.43, *p* < 0.001). When controlling for these additional factors, the greatest difference in multimorbidity between those with and without SMI was in those aged 18 to 29 (Table 2).

**Fig 2:**
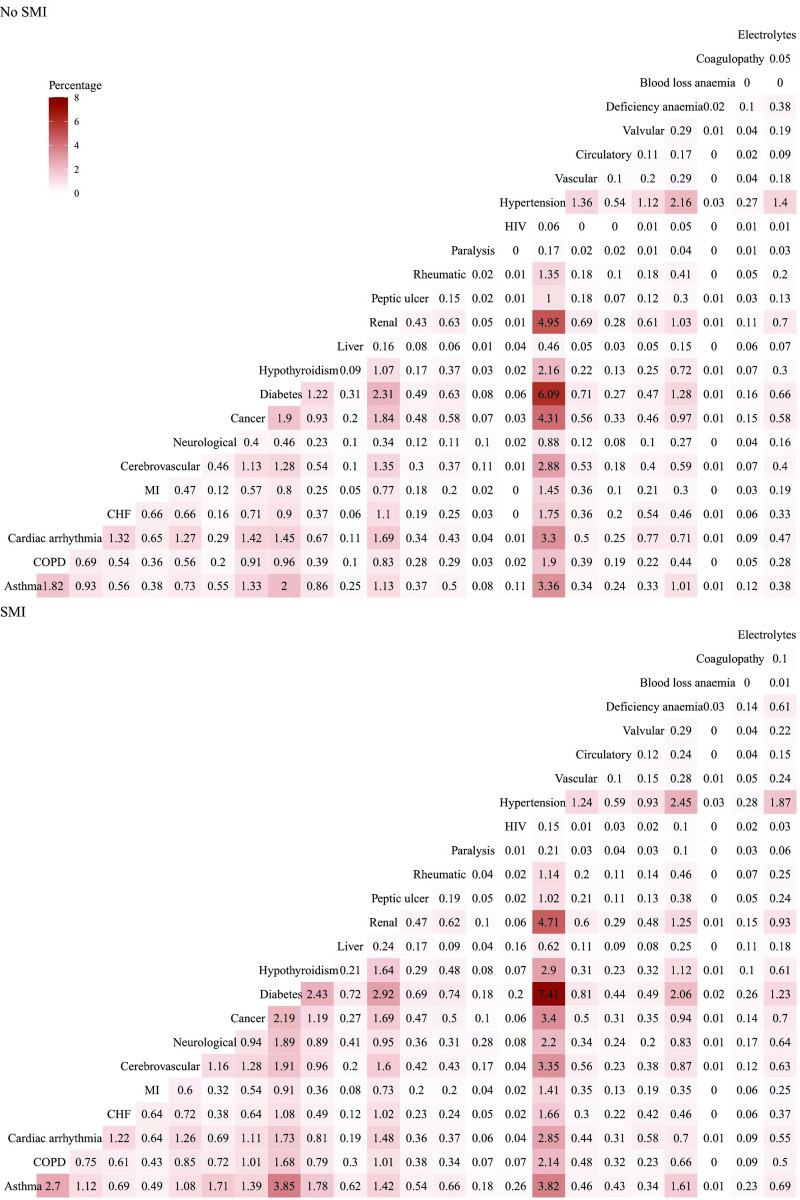
Percentage of patients with each combination of physical health condition pairs in patients with SMI and matched comparators. CHF, congestive heart failure; circulatory, pulmonary circulation disorders; COPD, chronic obstructive pulmonary disease; electrolytes, fluid and electrolyte disorders; HIV, human immunodeficiency virus; MI, myocardial infarction; paralysis, paresis/paralysis; rheumatic, rheumatic and collagen disease; SMI, severe mental illness; vascular, peripheral vascular disease.

The most common physical health conditions were hypertension, asthma, and diabetes in both SMI and comparator cohorts and when stratified by SMI diagnosis (Table 3). The most common multimorbidity pairs were the same in both cohorts: hypertension and diabetes (SMI: 7.41%; no SMI: 6.09%) followed by hypertension and renal disease (SMI: 4.71%; no SMI: 4.95%; Fig 2).

**Table 3:** Prevalence of physical health conditions.

	Prevalence, *n* (%)					Unadjusted OR (95% CI)				Demographic aOR^a^ (95% CI)				Demographic and risk aOR^b^ (95% CI)			
	No SMI	SMI	Schiz^c^	Bipolar	Other	SMI	Schiz^c^	Bipolar	Other	SMI	Schiz^c^	Bipolar	Other	SMI	Schiz^c^	Bipolar	Other
Asthma	41,817 (15.22)	12,759 (18.55)	2,352 (15.65)	5,131 (21.01)	5,276 (17.99)	1.27 (1.24 to 1.30) *p* < 0.001	1.03 (0.99 to 1.08) *p* = 0.16	1.48 (1.43 to 1.53) *p* < 0.001	1.22 (1.18 to 1.26) *p* < 0.001	1.28 (1.25 to 1.30) *p* < 0.001	1.07 (1.02 to 1.12) *p* = 0.003	1.45 (1.40 to 1.49) *p* < 0.001	1.24 (1.20 to 1.28) *p* < 0.001	1.06 (1.03 to 1.08) *p* < 0.001	0.87 (0.83 to 0.91) *p* < 0.001	1.17 (1.13 to 1.21) *p* < 0.001	1.06 (1.02 to 1.09) *p* < 0.001
COPD	10,187 (3.71)	3,795 (5.52)	781 (5.20)	1,426 (5.84)	1,588 (5.41)	1.52 (1.46 to 1.58) *p* < 0.001	1.42 (1.32 to 1.53) *p* < 0.001	1.61 (1.52 to 1.70) *p* < 0.001	1.49 (1.41 to 1.57) *p* < 0.001	1.61 (1.55 to 1.67) *p* < 0.001	1.81 (1.67 to 1.95) *p* < 0.001	1.90 (1.79 to 2.01) *p* < 0.001	1.34 (1.27 to 1.42) *p* < 0.001	1.21 (1.16 to 1.26) *p* < 0.001	1.27 (1.17 to 1.37) *p* < 0.001	1.36 (1.28 to 1.45) *p* < 0.001	1.07 (1.01 to 1.14) *p* = 0.02
Cardiac arrhythmia	14,465 (5.27)	3,666 (5.33)	546 (3.63)	1,177 (4.82)	1,943 (6.62)	(0.98 to 1.05) *p* = 0.50	0.68 (0.62 to 0.74) *p* < 0.001	0.91 (0.86 to 0.97) *p* = 0.003	1.28 (1.22 to 1.34) *p* < 0.001	1.06 (1.02 to 1.10) *p* = 0.003	0.91 (0.83 to 0.99) *p* = 0.03	1.11 (1.04 to 1.18) *p* = 0.002	1.09 (1.03 to 1.15) *p* = 0.002	1.00 (0.96 to 1.04) *p* = 0.91	0.86 (0.78 to 0.94) *p* < 0.001	0.99 (0.92 to 1.05) *p* = 0.67	1.06 (1.00 to 1.11) *p* = 0.04
Congestive heart failure	7,073 (2.57)	1,894 (2.75)	291 (1.94)	585 (2.40)	1,018 (3.47)	1.07 (1.02 to 1.13) *p* = 0.009	0.75 (0.66 to 0.84) *p* < 0.001	0.93 (0.85 to 1.01) *p* = 0.09	1.36 (1.27 to 1.45) *p* < 0.001	1.14 (1.08 to 1.20) *p* < 0.001	1.06 (0.93 to 1.20) *p* = 0.38	1.27 (1.16 to 1.38) *p* < 0.001	1.10 (1.03 to 1.18) *p* = 0.008	1.07 (1.01 to 1.13) *p* = 0.02	0.98 (0.87 to 1.11) *p* = 0.79	1.11 (1.01 to 1.21) *p* = 0.03	1.08 (1.00 to 1.16) *p* = 0.05
Myocardial infarction	6,237 (2.27)	1,593 (2.32)	254 (1.69)	461 (1.89)	878 (2.99)	1.02 (0.97 to 1.08) *p* = 0.45	0.74 (0.65 to 0.84) *p* < 0.001	0.83 (0.75 to 0.91) *p* < 0.001	1.33 (1.24 to 1.43) *p* < 0.001	1.07 (1.01 to 1.14) *p* = 0.02	0.96 (0.84 to 1.09) *p* = 0.52	1.02 (0.92 to 1.12) *p* = 0.71	1.15 (1.06 to 1.24) *p* < 0.001	1.00 (0.94 to 1.06) *p* = 0.89	0.87 (0.76 to 0.99) *p* = 0.03	0.89 (0.80 to 0.98) *p* = 0.02	1.12 (1.04 to 1.21) *p* = 0.004
Cerebrovascular disease	12,049 (4.39)	4,071 (5.92)	597 (3.97)	1,364 (5.59)	2,110 (7.19)	1.37 (1.32 to 1.42) *p* < 0.001	0.90 (0.83 to 0.98) *p* = 0.02	1.29 (1.22 to 1.37) *p* < 0.001	1.69 (1.61 to 1.77) *p* < 0.001	1.51 (1.45 to 1.57) *p* < 0.001	1.26 (1.15 to 1.38) *p* < 0.001	1.70 (1.60 to 1.81) *p* < 0.001	1.47 (1.40 to 1.55) *p* < 0.001	1.40 (1.35 to 1.46) *p* < 0.001	1.16 (1.06 to 1.27) *p* < 0.001	1.52 (1.43 to 1.62) *p* < 0.001	1.41 (1.34 to 1.49) *p* < 0.001
Neurological disease	8,071 (2.94)	5,509 (8.01)	1,079 (7.18)	1,765 (7.23)	2,665 (9.08)	2.88 (2.78 to 2.98) *p* < 0.001	2.56 (2.39 to 2.73) *p* < 0.001	2.57 (2.44 to 2.71) *p* < 0.001	3.30 (3.15 to 3.45) *p* < 0.001	2.92 (2.82 to 3.03) *p* < 0.001	2.80 (2.62 to 2.99) *p* < 0.001	2.62 (2.49 to 2.77) *p* < 0.001	3.22 (3.08 to 3.38) *p* < 0.001	2.63 (2.54 to 2.73) *p* < 0.001	2.53 (2.36 to 2.7) *p* < 0.001	2.34 (2.22 to 2.48) *p* < 0.001	2.92 (2.79 to 3.06) *p* < 0.001
Cancer	23,994 (8.74)	5,488 (7.98)	841 (5.60)	2,069 (8.79)	2,578 (8.79)	0.91 (0.88 to 0.93) *p* < 0.001	0.62 (0.58 to 0.66) *p* < 0.001	0.97 (0.92 to 1.01) *p* = 0.16	1.01 (0.96 to 1.05) *p* = 0.76	0.92 (0.89 to 0.95) *p* < 0.001	0.80 (0.74 to 0.86) *p* < 0.001	1.11 (1.05 to 1.16) *p* < 0.001	0.85 (0.81 to 0.89) *p* < 0.001	0.89 (0.86 to 0.92) *p* < 0.001	0.77 (0.71 to 0.83) *p* < 0.001	1.03 (0.98 to 1.08) *p* = 0.27	0.83 (0.79 to 0.87) *p* < 0.001
Diabetes	29,836 (10.86)	12,028 (17.49)	2,942 (19.58)	4,289 (17.56)	4,797 (16.35)	1.74 (1.70 to 1.78) *p* < 0.001	2.00 (1.92 to 2.08) *p* < 0.001	1.75 (1.69 to 1.81) *p* < 0.001	1.60 (1.55 to 1.66) *p* < 0.001	1.81 (1.77 to 1.86) *p* < 0.001	2.11 (2.02 to 2.21) *p* < 0.001	2.05 (1.98 to 2.13) *p* < 0.001	1.50 (1.45 to 1.56) *p* < 0.001	1.47 (1.43 to 1.51) *p* < 0.001	1.66 (1.59 to 1.74) *p* < 0.001	1.51 (1.45 to 1.57) *p* < 0.001	1.34 (1.30 to 1.39) *p* < 0.001
Hypothyroidism	13,229 (4.82)	5,468 (7.95)	794 (5.28)	2,667 (10.92)	2,007 (6.84)	1.71 (1.65 to 1.76) *p* < 0.001	1.10 (1.02 to 1.19) *p* = 0.009	2.42 (2.32 to 2.53) *p* < 0.001	1.45 (1.38 to 1.52) *p* < 0.001	1.80 (1.74 to 1.87) *p* < 0.001	1.50 (1.39 to 1.62) *p* < 0.001	2.58 (2.47 to 2.71) *p* < 0.001	1.35 (1.28 to 1.42) *p* < 0.001	1.68 (1.62 to 1.74) *p* < 0.001	1.39 (1.28 to 1.50) *p* < 0.001	2.28 (2.17 to 2.39) *p* < 0.001	1.32 (1.25 to 1.39) *p* < 0.001
Liver disease	4,061 (1.48)	1,973 (2.87)	460 (3.06)	588 (2.41)	925 (3.15)	1.97 (1.86 to 2.08) *p* < 0.001	2.10 (1.91 to 2.32) *p* < 0.001	1.64 (1.51 to 1.79) *p* < 0.001	2.17 (2.02 to 2.33) *p* < 0.001	1.95 (1.85 to 2.06) *p* < 0.001	1.98 (1.80 to 2.19) *p* < 0.001	1.74 (1.60 to 1.90) *p* < 0.001	2.09 (1.95 to 2.25) *p* < 0.001	1.07 (1.01 to 1.14) *p* = 0.02	1.10 (0.99 to 1.22) *p* = 0.08	0.98 (0.90 to 1.08) *p* = 0.74	1.12 (1.04 to 1.22) *p* = 0.004
Renal disease	18,357 (6.68)	5,351 (7.78)	893 (5.94)	1,949 (7.98)	2,509 (8.55)	1.18 (1.14 to 1.22) *p* < 0.001	0.88 (0.82 to 0.95) *p* < 0.001	1.21 (1.15 to 1.27) *p* < 0.001	1.31 (1.25 to 1.36) *p* < 0.001	1.27 (1.23 to 1.32) *p* < 0.001	1.23 (1.14 to 1.33) *p* < 0.001	1.63 (1.55 to 1.72) *p* < 0.001	1.07 (1.02 to 1.13) *p* = 0.004	1.20 (1.16 to 1.25) *p* < 0.001	1.17 (1.08 to 1.26) *p* < 0.001	1.42 (1.34 to 1.50) *p* < 0.001	1.07 (1.02 to 1.13) *p* = 0.006
Peptic ulcer	5,758 (2.09)	1,721 (2.50)	249 (1.66)	582 (2.38)	890 (3.03)	1.20 (1.14 to 1.27) *p* < 0.001	0.79 (0.70 to 0.90) *p* < 0.001	1.15 (1.05 to 1.25) *p* = 0.002	1.47 (1.37 to 1.58) *p* < 0.001	1.24 (1.17 to 1.31) *p* < 0.001	0.89 (0.78 to 1.02) *p* = 0.09	1.32 (1.21 to 1.45) *p* < 0.001	1.32 (1.23 to 1.43) *p* < 0.001	1.05 (0.99 to 1.12) *p* = 0.07	0.74 (0.65 to 0.84) *p* < 0.001	1.10 (1.01 to 1.20) *p* = 0.03	1.16 (1.07 to 1.25) *p* < 0.001
Rheumatic/collagen diseases	7,562 (2.75)	1,835 (2.67)	231 (1.54)	708 (2.90)	896 (3.05)	0.97 (0.92 to 1.02) *p* = 0.22	0.55 (0.48 to 0.63) *p* < 0.001	1.05 (0.98 to 1.14) *p* = 0.18	1.11 (1.04 to 1.19) *p* = 0.003	0.98 (0.93 to 1.04) *p* = 0.56	0.71 (0.62 to 0.81) *p* < 0.001	1.15 (1.06 to 1.24) *p* < 0.001	0.97 (0.91 to 1.05) *p* = 0.46	0.94 (0.89 to 0.99) *p* = 0.02	0.67 (0.59 to 0.77) *p* < 0.001	1.05 (0.97 to 1.14) *p* = 0.22	0.96 (0.89 to 1.03) *p* = 0.25
Paresis/paralysis	1,246 (0.45)	608 (0.88)	92 (0.61)	221 (0.90)	295 (1.01)	1.96 (1.78 to 2.16) *p* < 0.001	1.35 (1.09 to 1.67) *p* = 0.005	2.00 (1.74 to 2.31) *p* < 0.001	2.23 (1.96 to 2.53) *p* < 0.001	1.96 (1.78 to 2.17) *p* < 0.001	1.43 (1.15 to 1.76) *p* = 0.01	2.04 (1.76 to 2.35) *p* < 0.001	2.16 (1.90 to 2.46) *p* < 0.001	1.91 (1.73 to 2.12) *p* < 0.001	1.38 (1.11 to 1.72) *p* = 0.003	1.96 (1.69 to 2.27) *p* < 0.001	2.13 (1.86 to 2.42) *p* < 0.001
HIV	1,769 (0.64)	752 (1.09)	117 (0.78)	299 (1.22)	336 (1.15)	1.71 (1.57 to 1.86) *p* < 0.001	1.21 (1.00 to 1.46) *p* = 0.05	1.91 (1.69 to 2.16) *p* < 0.001	1.79 (1.59 to 2.01) *p* < 0.001	1.66 (1.52 to 1.81) *p* < 0.001	1.14 (0.94 to 1.38) *p* = 0.18	1.82 (1.61 to 2.07) *p* < 0.001	1.79 (1.59 to 2.02) *p* < 0.001	1.25 (1.14 to 1.37) *p* < 0.001	0.85 (0.70 to 1.03) *p* = 0.10	1.38 (1.21 to 1.57) *p* < 0.001	1.34 (1.19 to 1.52) *p* < 0.001
Hypertension	56,327 (20.51)	13,954 (20.29)	2,494 (16.60)	5,040 (20.64)	6,420 (21.89)	0.99 (0.97 to 1.01) *p* = 0.20	0.77 (0.74 to 0.81) *p* < 0.001	1.01 (0.98 to 1.04) *p* = 0.62	1.09 (1.05 to 1.12) *p* < 0.001	0.99 (0.96 to 1.01) *p* = 0.29	0.88 (0.83 to 0.92) *p* < 0.001	1.17 (1.13 to 1.21) *p* < 0.001	0.90 (0.87 to 0.94) *p* < 0.001	0.80 (0.78 to 0.82) *p* < 0.001	0.70 (0.66 to 0.74) *p* < 0.001	0.85 (0.82 to 0.88) *p* < 0.001	0.80 (0.78 to 0.83) *p* < 0.001
Peripheral vascular disease	5,502 (2.00)	1,473 (2.14)	224 (1.49)	503 (2.06)	746 (2.54)	1.07 (1.01 to 1.13) *p* = 0.02	0.74 (0.65 to 0.85) *p* < 0.001	1.03 (0.94 to 1.13) *p* = 0.55	1.28 (1.18 to 1.38) *p* < 0.001	1.13 (1.06 to 1.20) *p* < 0.001	0.96 (0.84 to 1.10) *p* = 0.58	1.29 (1.17 to 1.42) *p* < 0.001	1.09 (1.01 to 1.18) *p* = 0.04	0.98 (0.92 to 1.04) *p* = 0.54	0.80 (0.69 to 0.91) *p* = 0.001	1.07 (0.97 to 1.18) *p* = 0.18	0.99 (0.92 to 1.08) *p* = 0.90
Pulmonary circulation disorders	2,842 (1.03)	1,050 (1.53)	172 (1.14)	383 (1.57)	495 (1.69)	1.48 (1.38 to 1.59) *p* < 0.001	1.11 (0.95 to 1.29) *p* = 0.20	1.52 (1.37 to 1.70) *p* < 0.001	1.64 (1.49 to 1.81) *p* < 0.001	1.52 (1.41 to 1.63) *p* < 0.001	1.39 (1.19 to 1.62) *p* < 0.001	1.70 (1.53 to 1.90) *p* < 0.001	1.45 (1.31 to 1.60) *p* < 0.001	1.32 (1.22 to 1.42) *p* < 0.001	1.19 (1.01 to 1.39) *p* = 0.04	1.38 (1.23 to 1.54) *p* < 0.001	1.33 (1.20 to 1.46) *p* < 0.001
Valvular disease	4,908 (1.79)	1,130 (1.64)	153 (1.02)	381 (1.56)	596 (2.03)	0.92 (0.86 to 0.98) *p* = 0.01	0.57 (0.48 to 0.66) *p* < 0.001	0.87 (0.78 to 0.97) *p* = 0.01	1.14 (1.05 to 1.24) *p* = 0.003	0.94 (0.88 to 1.01) *p* = 0.08	0.73 (0.62 to 0.85) *p* < 0.001	1.03 (0.93 to 1.15) *p* = 0.59	0.97 (0.88 to 1.05) *p* = 0.44	0.91 (0.85 to 0.97) *p* = 0.004	0.70 (0.59 to 0.83) *p* < 0.001	0.95 (0.85 to 1.05) *p* = 0.31	0.95 (0.87 to 1.04) *p* = 0.27
Deficiency anaemia	14,320 (5.21)	4,786 (6.96)	891 (5.93)	1,693 (6.93)	2,202 (7.51)	1.36 (1.31 to 1.41) *p* < 0.001	1.15 (1.07 to 1.23) *p* < 0.001	1.35 (1.29 to 1.43) *p* < 0.001	1.48 (1.41 to 1.55) *p* < 0.001	1.37 (1.32 to 1.41) *p* < 0.001	1.33 (1.24 to 1.43) *p* < 0.001	1.38 (1.31 to 1.46) *p* < 0.001	1.37 (1.30 to 1.44) *p* < 0.001	1.30 (1.25 to 1.34) *p* < 0.001	1.27 (1.18 to 1.36) *p* < 0.001	1.27 (1.20 to 1.34) *p* < 0.001	1.33 (1.27 to 1.40) *p* < 0.001
Blood loss anaemia	185 (0.07)	43 (0.06)	11 (0.07)	14 (0.06)	18 (0.06)	0.93 (0.67 to 1.29) *p* = 0.66	1.09 (0.59 to 2.00) *p* = 0.79	0.85 (0.49 to 1.47) *p* = 0.56	0.91 (0.56 to 1.48) *p* = 0.71	0.89 (0.64 to 1.25) *p* = 0.51	1.17 (0.63 to 2.16) *p* = 0.62	0.87 (0.50 to 1.50) *p* = 0.62	0.80 (0.49 to 1.29) *p* = 0.36	0.86 (0.62 to 1.21) *p* = 0.40	1.11 (0.60 to 2.06) *p* = 0.74	0.82 (0.47 to 1.42) *p* = 0.48	0.79 (0.49 to 1.29) *p* = 0.35
Coagulopathy	1,795 (0.65)	614 (0.89)	100 (0.67)	224 (0.92)	290 (0.99)	1.37 (1.25 to 1.50) *p* < 0.001	1.02 (0.83 to 1.25) *p* = 0.86	1.41 (1.22 to 1.62) *p* < 0.001	1.52 (1.34 to 1.72) *p* < 0.001	1.37 (1.25 to 1.50) *p* < 0.001	1.07 (0.87 to 1.31) *p* = 0.51	1.47 (1.28 to 1.69) *p* < 0.001	1.43 (1.26 to 1.62) *p* < 0.001	1.16 (1.06 to 1.28) *p* = 0.002	0.91 (0.74 to 1.12) *p* = 0.38	1.21 (1.05 to 1.39) *p* = 0.01	1.25 (1.10 to 1.42) *p* < 0.001
Fluid and electrolyte disorders	5,341 (1.94)	2,309 (3.36)	355 (2.36)	863 (3.53)	1,091 (3.72)	1.75 (1.67 to 1.84) *p* < 0.001	1.22 (1.09 to 1.36) *p* < 0.001	1.85 (1.72 to 1.99) *p* < 0.001	1.95 (1.82 to 2.08) *p* < 0.001	1.85 (1.76 to 1.95) *p* < 0.001	1.59 (1.42 to 1.77) *p* < 0.001	2.28 (2.11 to 2.46) *p* < 0.001	1.68 (1.57 to 1.80) *p* < 0.001	1.63 (1.54 to 1.71) *p* < 0.001	1.39 (1.24 to 1.56) *p* < 0.001	1.89 (1.75 to 2.04) *p* < 0.001	1.54 (1.44 to 1.65) *p* < 0.001

^a^Demographic-adjusted: adjusted for age, sex, ethnicity, and region.

^b^Demographic and risk adjusted: adjusted for age, sex, ethnicity, and region and, additionally, for smoking status, BMI category, alcohol misuse, and drug misuse.

^c^Schizophrenia.

aOR, adjusted odds ratio; BMI, body mass index; CI, confidence interval; COPD, chronic obstructive pulmonary disease; HIV, human immunodeficiency virus; OR, odds ratio; SMI, severe mental illness.

**Fig 3:**
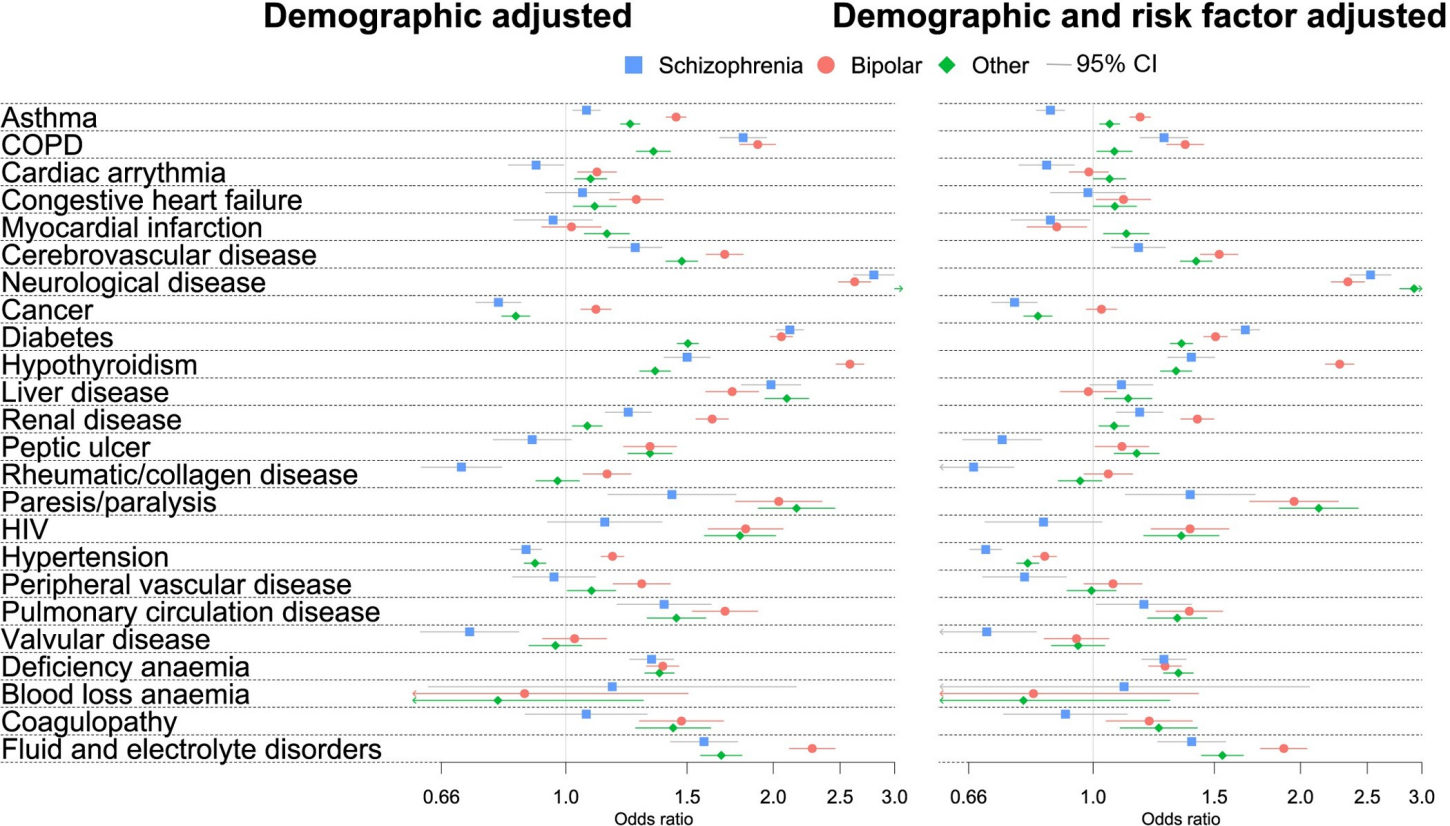
ORs of physical health conditions in those with SMI compared to the comparator population. CI, confidence interval; COPD, chronic obstructive pulmonary disease; HIV, human immunodeficiency virus; OR, odds ratio; SMI, severe mental illness.

When adjusting for age, sex, ethnicity, and region, patients with SMI had greater odds of recorded diagnoses of 19 out of 24 diseases (Fig 3, Table 3). ORs were particularly high for neurological disease (aOR: 2.92; 95% CI: 2.82 to 3.03, *p* < 0.001), paralysis or paresis (aOR: 1.96; 95% CI: 1.78 to 2.17, *p* < 0.001). and liver disease (aOR: 1.95; 95% CI: 1.85 to 2.06, *p* < 0.001). When stratified by SMI diagnosis, patients with schizophrenia had lower odds of recorded cardiac arrhythmia, cancer, valvular disease, rheumatoid and collagen disease, and hypertension than the comparator population, while patients with bipolar disorder had particularly high rates of hypothyroidism and fluid and electrolyte disorders (Table 3).

Patients with SMI had more health risk factors than the comparator cohort (Table 1). Obesity was particularly prevalent in those with a diagnosis of bipolar disorder (42.68%), while smoking was most prevalent in those with schizophrenia (53.75%), and alcohol and drug misuse was most prevalent in those with a diagnosis of other psychoses (14.11% and 14.68%, respectively). After adjustment for these risk factors, the ORs for all diseases in the SMI cohort reduced, in particular for liver disease, HIV, COPD, diabetes, and neurological disease (Fig 3, Table 3).

### Clustering of physical health conditions and multimorbidity profiles

In MCA of patients with physical health multimorbidity (SMI cohort: 23,382 (33.99%), comparators: 70,003 (25.48%)), 16 dimensions were required to explain 70% of the variance of physical health conditions in both the SMI cohort and comparator cohort. The first 2 dimensions in MCA had similar disease profiles (S1 Fig).

**Fig 4:**
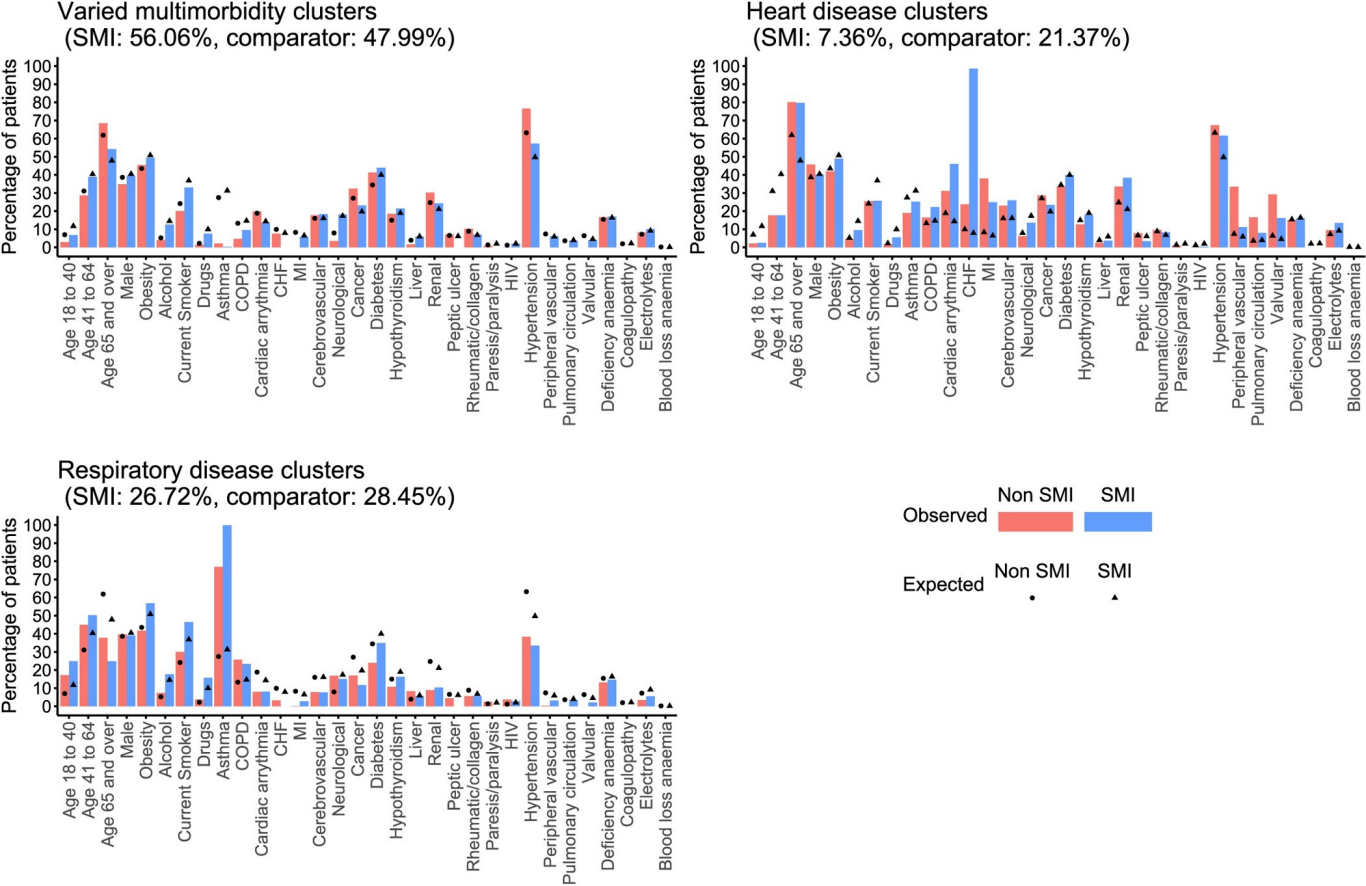
Prevalence of physical health conditions, health risk factors, and demographics in the SMI and comparator cohorts for the 3 most common multimorbidity clusters. *Heart disease in the comparator population is the pooled prevalence across 3 heart disease clusters. CHF, congestive heart failure; COPD, chronic obstructive pulmonary disease; electrolytes, fluid and electrolyte disorders; HIV, human immunodeficiency virus; MI, myocardial infarction.

We identified 7 profiles of physical health multimorbidity in both those with and without SMI. The largest patient group in both populations (56.06% of the SMI population and 47.99% of the comparator population) consisted of patients with varied multimorbidity (Table 4, Fig 4). In those with SMI, a second cluster consisted of patients with a high prevalence of heart disease (7.36%), while 3 distinct heart disease clusters were found in those without SMI: one of predominantly valvular disease (5.78%), one of pulmonary circulatory disease (3.55%), and one of myocardial infarction and peripheral vascular disease (12.03%). All of these clusters were characterised by older age, and varied multimorbidity, heart disease, and valvular disease had low prevalence of health risk factors. We identified a cluster of respiratory disease in both cohorts (26.72% in those with SMI and 28.45% in comparators) associated with younger age and a higher prevalence of smoking or substance misuse and 2 small clusters of patients with blood loss anaemia and coagulopathy (Table 4).

**Table 4:** Profiles of multimorbidity identified in K-means cluster analysis.

SMI (*n* = 23,382)			No SMI (*n* = 70,003)		
Cluster description	Observed in cluster/expected* >1.2 or <0.8	High frequency variables, *n* (%)	Cluster description	Observed in cluster/expected* >1.2 or <0.8	High frequency variables, *n* (%)
**Varied multimorbidity**					
***n* (%):** 13,107 (56.06) **Median (IQR):** Conditions: 3 (2 to 3) Age: 67.63 (54.75 to 80.51)	**Chronic diseases** High: None Low: Congestive heart failure (0.0), peptic ulcer (0.0), blood loss anaemia (0.0), paresis/paralysis (0.0), coagulopathy (0.0), asthma (0.01), and COPD (0.65) **Health risk factors and demographics** High: None Low: Age 18 to 40 (0.58) and drug misuse (0.76) **SMI:** None	**Cluster contains > = 70% of all patients with this variable:** None **> = 70% of patients in this cluster have this variable:** None	***n* (%):** 33,610 (47.99) **Median (IQR):** Conditions: 3 (2 to 4) Age: 74.27 (61.89 to 85.00)	**Chronic diseases** High: Hypothyroidism (1.24), renal disease (1.24), and hypertension (1.21) Low: Myocardial infarction (0.0), blood loss anaemia (0.0), coagulopathy (0.0), peripheral vascular disease (0.00), pulmonary circulatory disorder (0.00), valvular disease (0.0), HIV (0.03), asthma (0.08), COPD (0.35), liver disease (0.42), neurological disease (0.44), paresis/paralysis (0.66), and congestive heart failure (0.77) **Health risk factors and demographics** High: None Low: Age 18 to 40 (0.41), drug misuse (0.67), underweight (0.73), and alcohol misuse (0.77)	**Cluster contains > = 70% of all patients with this variable:** None **> = 70% of patients in this cluster have this variable:** Hypertension (76.61)
**Heart disease**					
**General heart disease**			**Valvular disease**		
***n* (%):** 1,720 (7.36) **Median (IQR):** Conditions: 5 (4 to 7) Age: 80.01 (68.92 to 87.28)	**Chronic diseases** High: Congestive heart failure (12.50), myocardial infarction (3.86), valvular disease (3.58), cardiac arrhythmia (3.21), pulmonary circulation disorders (2.01), peripheral vascular disease (1.91), renal disease (1.82), cerebrovascular disease (1.61), COPD (1.52), fluid and electrolyte disorders (1.47), hypertension (1.24), and rheumatic/collagen disease (1.22) Low: Blood loss anaemia (0.0), coagulopathy (0.0), paresis/paralysis (0.0), HIV (0.20), peptic ulcer (0.56), liver disease (0.62), neurological disease (0.78), and asthma (0.80) **Health risk factors and demographics** High: age 65+ (1.67) Low: Age 18 to 40 (0.21), age 41 to 64 (0.44), drug misuse (0.56), alcohol misuse (0.65), and current smoker (0.70) **SMI:** None	**Cluster contains > = 70% of all patients with this variable:** Congestive heart failure (91.93), **> = 70% of patients in this cluster have this variable:** Congestive heart failure (98.66)	***n* (%):** 4,049 (5.78) **Median (IQR):** Conditions: 5 (3 to 6) Age: 80.93 (69.95 to 88.00)	**Chronic diseases** High: Valvular disease (15.54), congestive heart failure (3.13), cardiac arrhythmia (2.46), fluid and electrolyte disorders (1.53), peripheral vascular disease (1.48), renal disease (1.45), cerebrovascular disease (1.41), and myocardial infarction (1.41) Low: Blood loss anaemia (0.0), coagulopathy (0.0), pulmonary circulation disorders (0.00), HIV (0.39), paresis/paralysis (0.54), liver disease (0.67), asthma (0.73), and neurological disease (0.74) **Health risk factors and demographics** High: Age 65+ (1.30) Low: Age 18 to 40 (0.56), age 41 to 64 (0.50) drug misuse (0.69), alcohol misuse (0.71), and current smoker (0.78)	**Cluster contains > = 70% of all patients with this variable:** Valvular disease (89.86) **> = 70% of patients in this cluster have this variable:** Valvular disease (100.00)
			**Pulmonary circulatory disorders**		
			***n* (%):** 2,485 (3.55) **Median (IQR):** Conditions: 4 (3 to 6) Age: 77.59 (65.00 to 86.00)	**Chronic diseases** High: Pulmonary circulation disorders (27.44), congestive heart failure (2.08), valvular disease (1.91), COPD (1.56), cardiac arrhythmia (1.43), peripheral vascular disease (1.37), fluid and electrolyte disorders (1.34), cancer (1.29), myocardial infarction (1.28), renal disease (1.23), cerebrovascular disease (1.23), paresis/paralysis (1.23), and rheumatic/collagen disease (1.21) Low: Blood loss anaemia (0.0), coagulopathy (0.0), and HIV (0.30) **Health risk factors and demographics** High: Age 65+ (1.21) Low: Age 41 to 64 (0.66) and age 18 to 40 (0.67)	**Cluster contains > = 70% of all patients with this variable:** Pulmonary circulation disorders (97.37) **> = 70% of patients in this cluster have this variable:** Pulmonary circulation disorders (100.00)
			**Myocardial infarctions and peripheral vascular disease**		
			***n* (%):** 8,424 (12.03) **Median (IQR):** Conditions: 4 (3 to 6) Age: 79.00 (68.83 to 86.73)	**Chronic diseases** High: Myocardial infarction (7.06), peripheral vascular disease (6.91), congestive heart failure (2.14), cerebrovascular disease (1.48), renal disease (1.35), cardiac arrhythmia (1.33), peptic ulcer (1.30), COPD (1.29), and fluid and electrolyte disorders (1.20) Low: Blood loss anaemia (0.0), coagulopathy (0.0), pulmonary circulation disorders (0.00), valvular disease (0.05), HIV (0.12), liver disease (0.56), asthma (0.60), paresis/paralysis (0.68), and neurological disease (0.71) **Health risk factors and demographics** High: Male (1.39), age 65+ (1.32), and current smoker (1.23) Low: Age 18 to 40 (0.09), age 41 to 64 (0.58), never smoker (0.65), drug misuse (0.65), and female (0.76)	**Cluster contains > = 70% of all patients with this variable:** Myocardial infarction (84.97), peripheral vascular disease (83.11) **> = 70% of patients in this cluster have this variable:** None
**Respiratory disease and HIV**					
***n* (%):** 6,248 (26.72) **Median (IQR):** Conditions: 3 (2 to 4) Age: 52.92 (41.20 to 65.00)	**Chronic diseases** High: Asthma (3.19), COPD (1.59), and HIV (1.38) Low: Blood loss anaemia (0.00), coagulopathy (0.0), paresis/paralysis (0.0), peptic ulcer (0.0), congestive heart failure (0.0), myocardial infarction (0.43), cerebrovascular disease (0.47), valvular disease (0.47), renal disease (0.49), peripheral vascular disease (0.54), cardiac arrhythmia (0.56), cancer (0.59), fluid and electrolyte disorders (0.61), and hypertension (0.67) **Health risk factors and demographics** High: Age 18 to 40 (2.11), drug misuse (1.58), current smoker (1.26), age 41 to 64 (1.25), and alcohol misuse (1.22) Low: Age 65+ (0.52), never smoked (0.70), and underweight (0.80) **SMI: None**	**Cluster contains > = 70% of all patients with this variable:** Asthma (85.20) **> = 70% of patients in this cluster have this variable:** Asthma (99.87)	***n* (%):** 19,921 (28.45) **Median (IQR):** Conditions: 2 (2 to 3) Age: 59.00 (47.00 to 73.00)	High: HIV (3.16), Asthma (2.80), neurological disease (2.13), liver disease (2.11), COPD (1.93), and paresis/paralysis (1.80) Low: Blood loss anaemia (0.00), coagulopathy (0.0), valvular disease (0.00), pulmonary circulation disorders (0.00), myocardial infarction (0.02), peripheral vascular disease (0.04), congestive heart failure (0.33), renal disease (0.36), cardiac arrhythmia (0.42), fluid and electrolyte disorders (0.47), cerebrovascular disease (0.49), hypertension (0.61), cancer (0.62), rheumatic/collagen disease (0.64), peptic ulcer (0.68), diabetes (0.69), and hypothyroidism (0.72) **Health risk factors and demographics** High: Age 18 to 40 (2.46), drug misuse (1.69), age 41 to 64 (1.45), underweight (1.46), alcohol misuse (1.42), and current smoker (1.24) Low: Age 65+ (0.61)	**Cluster contains > = 70% of all patients with this variable:** HIV (89.89), asthma (79.59) **> = 70% of patients in this cluster have this variable:** Asthma (76.90)
**Blood loss anaemia**					
***n* (%):** 35 (0.15) **Median (IQR):** Conditions: 4 (3 to 5.5) Age: 66.00 (55.00 to 80.37)	**Chronic diseases** High: Blood loss anaemia (668.06), deficiency anaemia (3.14), coagulopathy (2.62), pulmonary circulation disorders (2.15), peripheral vascular disease (1.94), cancer (1.45), paresis/paralysis (1.42), peptic ulcer (1.40), rheumatic/collagen disease (1.26), fluid and electrolyte disorders (1.25), and renal disease (1.22) Low: HIV (0.00), myocardial infarction (0.44), liver disease (0.48), COPD (0.58), valvular disease (0.63), congestive heart failure (0.72), and asthma (0.73) **Health risk factors and demographics** High: Underweight (8.20), ex-smoker (1.55), and female (1.20) Low: Age 18 to 40 (0.49), never smoked (0.50), drug misuse (0.57), alcohol misuse (0.59), current smoker (0.70), male (0.71), and normal weight (0.73) **SMI:** High: Schizophrenia (1.25)	**> = 70% of patients in this cluster have this variable:** Blood loss anaemia (100.00) **> = 70% of patients in this cluster have this variable:** Blood loss anaemia (100.00)	***n* (%):** 153 (0.22) **Median (IQR):** Conditions: 4 (2 to 5) Age: 63.00 (52.37 to 83.72)	**Chronic diseases** High: Blood loss anaemia (457.73), coagulopathy (2.94), deficiency anaemia (2.75), valvular disease (2.13), HIV (1.63), and peptic ulcer (1.59) Low: Paresis/paralysis (0.0), COPD (0.44), hypothyroidism (0.65), cardiac arrhythmia (0.66), neurological disease (0.74), cancer (0.75), diabetes (0.78), cerebrovascular disease (0.78), and hypertension (0.79) **Health risk factors and demographics** High: Age 41 to 64 (1.53) and female (1.40) Low: Underweight (0.00), alcohol misuse (0.25), male (0.37), and age 65+ (0.75)	**> = 70% of patients in this cluster have this variable:** Blood loss anaemia (100.00) **> = 70% of patients in this cluster have this variable:** Blood loss anaemia (100.00), female (85.62)
**Coagulopathy**					
***n* (%):** 493 (2.11) **Median (IQR):** Conditions: 4 (2 to 5) Age: 59.00 (47.59 to 74.00)	**Chronic diseases** High: Coagulopathy (45.94), liver disease (2.41), HIV (1.49), fluid and electrolyte disorders (1.49), rheumatic/collagen disease (1.28), myocardial infarction (1.25), valvular disease (1.25), neurological disease (1.24), peripheral vascular disease (1.24), and pulmonary circulation disorders (1.22) Low: Blood loss anaemia (0.00) and hypothyroidism (0.75) **Health risk factors and demographics** High: Alcohol misuse (1.59), drug misuse (1.51), and age 18 to 40 (1.36) Low: None **SMI:** None	**> = 70% of patients in this cluster have this variable:** Coagulopathy (96.86) **> = 70% of patients in this cluster have this variable:** Coagulopathy (100.00)	***n* (%):** 1,391 (1.99) **Median (IQR):** Conditions: 4 (2 to 5) Age: 68.00 (52.68 to 81.94)	**Chronic diseases** High: Coagulopathy (50.02), liver disease (2.83), HIV (1.74), fluid and electrolyte disorders (1.46), deficiency anaemia (1.24), and pulmonary circulation disorders (1.22) Low: Blood loss anaemia (0.0), COPD (0.74), paresis/paralysis (0.79), and myocardial infarction (0.80) **Health risk factors and demographics** High: Drug misuse (1.83), age 18 to 40 (1.72), and alcohol misuse (1.62) Low: None	**> = 70% of patients in this cluster have this variable:** Coagulopathy (99.36) **> = 70% of patients in this cluster have this variable:** Coagulopathy (100.00)
**Clusters specific to SMI**					
**Peptic ulcer disease**					
***n* (%):** 1,313 (5.62) **Median (IQR):** Conditions: 4 (3 to 5) Age: 70.00 (57.97 to 82.00)	**Chronic diseases** High: Peptic ulcer (16.32), peripheral vascular disease (1.54), and liver disease (1.25) Low: Paresis/paralysis (0.0), blood loss anaemia (0.0), coagulopathy (0.0), HIV (0.30), hypothyroidism (0.65), congestive heart failure (0.76), diabetes (0.79), and asthma (0.80) **Health risk factors and demographics** High: Underweight (2.19), alcohol misuse (1.56), male (1.30), and age 65+ (1.26) Low: Never smoked (0.76) and female (0.80) **SMI:** Low: Schizophrenia (0.79)	**> = 70% of patients in this cluster have this variable:** Peptic ulcer (91.63) **> = 70% of patients in this cluster have this variable:** Peptic ulcer (100.00)			
**Paresis/paralysis**					
***n* (%):** 466 (1.99) **Median (IQR):** Conditions: 3 (2 to 4) Age: 54.93 (47.59 to 74.00)	**Chronic diseases** High: Paresis/paralysis (49.54), neurological disease (2.32), cerebrovascular disease (1.44), and coagulopathy (1.38) Low: Blood loss anaemia (0.0), peripheral vascular disease (0.51), hypothyroidism (0.53), cardiac arrhythmia (0.57), rheumatic/collagen disease (0.57), COPD (0.59), hypertension (0.60), diabetes (0.64), liver disease (0.65), renal disease (0.65), valvular disease (0.66), congestive heart failure (0.71), and HIV (0.74) **Health risk factors and demographics** High: Age 18 to 40 (1.92), never smoked (1.40), normal weight (1.36), and male (1.27) Low: Underweight (0.62), age 65+ (0.68), and ex-smoker (0.80) **SMI:** Low: Schizophrenia (0.75)	**> = 70% of patients in this cluster have this variable:** Paresis/paralysis (98.72) **> = 70% of patients in this cluster have this variable:** Paresis/paralysis (100.00)			

Expected: the prevalence in the multimorbidity SMI or comparator cohort.

COPD, chronic obstructive pulmonary disease; HIV, human immunodeficiency virus; SMI, severe mental illness.

Finally, we identified 2 small clusters unique to the SMI population and 2 unique to comparators, comprising 7.61% (*n* = 1,779) of the multimorbid patients in total. One cluster consisted of patients with peptic ulcer disease and the other paresis or paralysis.

When we included health risk factors in the cluster analysis, 6 of the 7 clusters were common to both those with and without SMI. The largest cluster we identified was a “general multimorbidity” cluster, accounting for 56.70% of the SMI and 70.30% of the comparator cohorts. We also identified a large cluster of in the SMI cohort defined by respiratory disease, a high prevalence of health risk factors, male sex, neurological disease, and liver disease (17.02%). In contrast, a similar cluster identified in the comparator cohort accounted for only 4.87% of patients.

### Sensitivity analyses

In sensitivity analysis, recoding missing ethnicity to “missing” did not alter the interpretation of disease prevalence (S1 Table), nor did using multiple imputation (S2 Table).

## Discussion

Our study investigated physical health conditions and multimorbidity in a large cohort of patients with SMI and matched comparators. Clustering of multimorbid health conditions was not dramatically different between those with and without SMI, despite higher prevalence of many physical health conditions in the SMI cohort. Patients with a diagnosis of SMI had a higher prevalence of multimorbidity, particularly in younger age groups.

### Patterns of physical health conditions and multimorbidity

To the best of our knowledge, our analysis is the first cluster analysis of multimorbidity in a large, representative cohort of patients with and without SMI and suggests that patients with SMI develop similar profiles of multimorbidity to the general population.

Similarities in physical health profiles of those with and without SMI were also apparent in individual and disease pair ranking, MCA, and cluster analysis. Two previous studies have found similarity in ranking the most frequently diagnosed conditions and pairs of conditions between those with and without SMI [1,28], and a hospital-based study of self-reported physical health conditions in 1,060 psychiatric patients and 837 members of the general population found similar profiles of multimorbidity between the 2 cohorts using latent class analysis [29].

Despite the similarities in clusters of diseases, those with SMI have a higher prevalence of physical health conditions, more risk factors for poor physical health and develop multimorbidity at a younger age. Health risk factors likely explain some of the higher risk of physical health conditions and multimorbidity in people with SMI. We found that including smoking status, BMI category, and alcohol and drug misuse in cluster analysis resulted in a higher proportion of patients in the SMI cohort being in a “health risk” cluster and that adjusting for these factors decreased the ORs of physical health conditions between SMI and comparator cohorts, particularly for liver disease, HIV, COPD, diabetes, and neurological disease.

In line with other studies [5], we found a higher prevalence of multimorbidity in women in both SMI and comparator cohorts. Higher prevalence of multimorbidity with increasing age is well described in the general population [7,20], but we found the largest differences between those with and without SMI in the younger age groups. This suggests that people with SMI develop multimorbidity earlier than the general population. A higher prevalence of multimorbidity in younger patients was also found in a study of multimorbidity in those with psychosis in lower- and middle-income countries [30]. At older ages, the similar prevalence of multimorbidity in those with and without SMI could be due to survivorship bias in the SMI cohort or due to the high background prevalence of multimorbidity at that age.

### Underascertainment of physical health conditions in patients with schizophrenia

We identified lower prevalence of a range of physical health conditions in patients with schizophrenia and also lower rates of multimorbidity in older age in this population. This is surprising given the observed high prevalence of smoking, obesity, and alcohol and drug misuse, and known side effects of antipsychotic medication [9]. Lower prevalence of cardiovascular disease [31–33] and cancer [31] have been reported in other studies using routine primary care data, and our study corroborates this finding using a matched comparator population and controlling for both demographic and health risk factors. Underreporting is likely not due to lack of contact between primary care physicians and patients with SMI, as in the UK, annual health checks in primary care have been recommended and incentivised in this patient group since 2004. This underreporting could reflect poor access to care, underdiagnosis, or diagnostic overshadowing in the schizophrenia population. There is evidence that those with schizophrenia are more likely to have physical health conditions recorded at the time of death [34,35], suggesting late and missed diagnoses in this population, with diagnoses at the time of death less likely to be subsequently recorded in primary care records.

### Strengths and limitations

To our knowledge, this study is the largest investigation of multimorbidity and clustering of physical health conditions in patients with SMI. A key strength of our study was the ability to adjust for smoking, BMI category, alcohol misuse, and drug misuse as risk factors for physical health conditions.

The large sample size of this study, and representativeness of data from CPRD [21,22], suggests that the results of this study are generalisable to the UK population with SMI. However, the population without SMI is likely not representative as they are matched to the SMI population and therefore share the population characteristics in terms of age, sex, and area of residence with the SMI population. The similarities between the populations may have diminished differences in disease prevalence between the 2 cohorts.

As with all studies using electronic health records, a limitation of this study is potential biases in recording variables. Surveillance bias may have resulted in higher disease detection in people with SMI, a population who may have more regular contact with the healthcare system, affording more opportunities for physical health conditions to be recorded. Furthermore, while the apparent underrecording of a range of physical health conditions in those with schizophrenia is a clinically important finding, it limits the interpretation of disease prevalence and multimorbidity clusters in this population.

There may be residual confounding due to missing information. For physical health conditions and risk factors, the absence of coding for a condition was assumed to mean absence of disease or risk factor. However, particularly for risk factors, some missingness may be due to lack of measurement or recording. Missing values for smoking status, ethnicity, and BMI were replaced in line with other primary care studies [25,26] and sensitivity analyses performed for ethnicity. For BMI, we were only able to include broad categories as some patients had BMI category recorded rather than a BMI value. BMI itself is an indirect measure of obesity, and its accuracy varies with age, gender, and ethnicity. This may have introduced biases into the analysis [36]. Alcohol misuse was based on medical code lists and did not account for the level of alcohol consumption, nor include patients that had consumption recorded without an accompanying alcohol misuse code. While we were unable to control for deprivation, patients were matched on primary care practice and therefore from a broadly comparable geographic area.

This study focused on physical health conditions ever diagnosed, which limits the study of temporality of diagnoses of SMI and physical health conditions. However, with both SMI and chronic physical health conditions, a prodromal stage or period of undiagnosed disease may occur, and, therefore, diagnosis dates may not give a clear indication of temporal association. Furthermore, previous studies have found higher prevalence of physical health problems [37–39] and health risk factors for physical health conditions such as smoking [40] and alcohol and drug misuse [41], prior to SMI diagnosis.

### Implications

The absence of large novel clusters of disease in those with SMI suggests that the same drivers of physical health conditions are at play in both those with and without SMI, and, therefore, research and service provision for patients with SMI should focus on the same disease clusters as in the general population. However, while much of the focus of multimorbidity in the general population has been on old age, our study found that the largest difference in multimorbidity was at younger ages. This highlights an unmet need in terms of interventions aimed at a younger cohort of multimorbid patients and demonstrating the importance of physical health checks in this population. We found a higher prevalence of obesity, smoking, drug and alcohol misuse in this population, and adjusting for these factors reduced the ORs of many diseases. This suggests that a focus on risk factor reduction would also reduce the incidence of physical health conditions in those with SMI. Interventions to modify these risk factors, for example, via smoking or alcohol cessation support [42,43], have been shown to be effective in people with SMI and need to be more widely available.

Further work is warranted to investigate the temporality of SMI and physical health condition diagnoses, and trajectories of multimorbidity in this population. The low prevalence of some physical health conditions in the schizophrenia cohort also requires further investigation, to elucidate the reasons for this finding. Finally, the relevance of the identified clusters to outcomes such as hospitalisation and mortality, both in patients with and without SMI, is an area for future research.

## Conclusions

We found that physical health conditions cluster in people with SMI in a similar manner to people without SMI. However, there is a higher prevalence of physical health conditions, physical health multimorbidity, and risk factors for poor physical health in those with SMI, and those with SMI may develop multimorbidity at a younger age. Therefore, while interventions aimed at the general population should also be applicable to those with SMI, there is a need for a greater focus on diseases of younger age, younger-age multimorbidity and of reduction of risk factors for poor physical health.

## Supporting information

S1 Checklist STROBE checklist. STROBE, Strengthening the Reporting of Observational Studies in Epidemiology.
(DOCX)

S1 Code Lists Code list for SMI and physical health conditions. SMI, severe mental illness.
(XLSX)

S1 Fig Pattern of variables in MCA in SMI and comparator cohorts. MCA, multiple correspondence analysis; SMI, severe mental illness.
(DOCX)

S1 Table ORs of physical health conditions in patients with missing ethnicity, compared to white ethnicity. OR, odds ratio.
(DOCX)

S2 Table aORs of physical health conditions after multiple imputation for missing ethnicity. aOR, adjusted odds ratio.
(DOCX)

## Abbreviations:

aOR: adjusted odds ratio
BMI: body mass index
CI: confidence intervals
COPD: chronic obstructive pulmonary disease
CPRD: Clinical Practice Research Datalink
HIV: Human immunodeficiency virus
IQR: interquartile range
MCA: multiple correspondence analysis
NICE: National Institute for Health Care and Excellence
OR: odds ratio
SMI: severe mental illness
STROBE: Strengthening the Reporting of Observational Studies in Epidemiology
