# Supplementary material for: Clustering of physical health multimorbidity in people with severe mental illness: An accumulated prevalence analysis of United Kingdom primary care data
Source: PLoS Med. 2022 Apr 20;19(4):e1003976. doi: 10.1371/journal.pmed.1003976 (PMC9067697; doi:10.1371/journal.pmed.1003976)
Supplement: S1 Fig — MCA, multiple correspondence analysis; SMI, severe mental illness. (DOCX) [file pmed.1003976.s003.docx]

#### Supplement 3: Pattern of variables in Multiple Correspondence Analysis in SMI and comparator cohorts


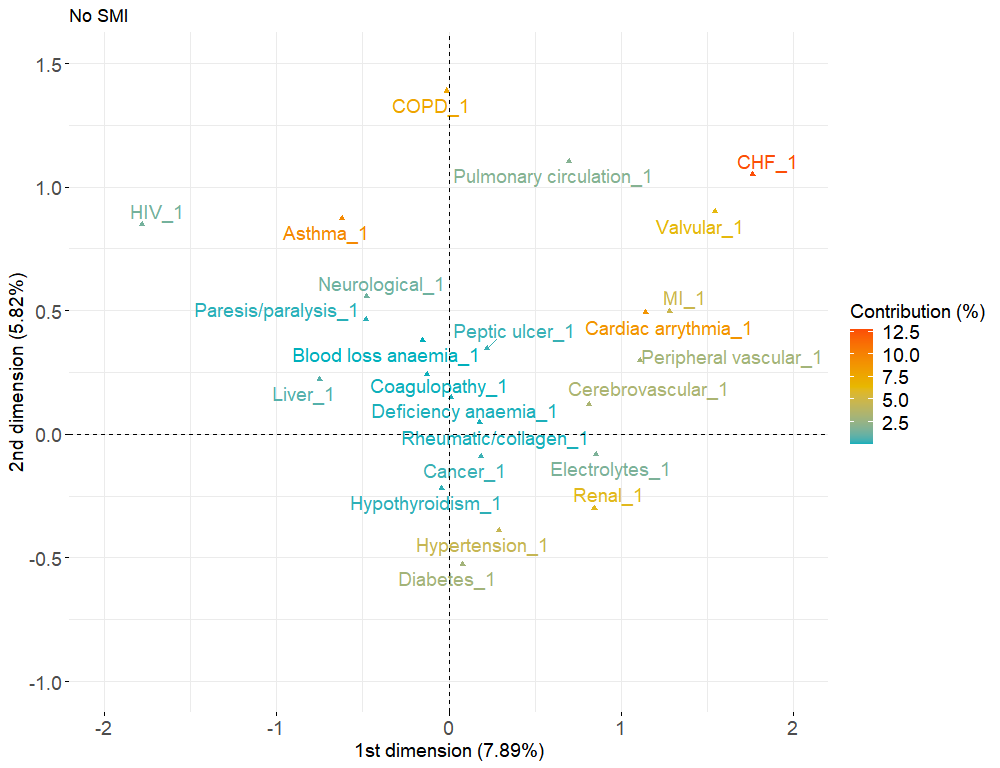


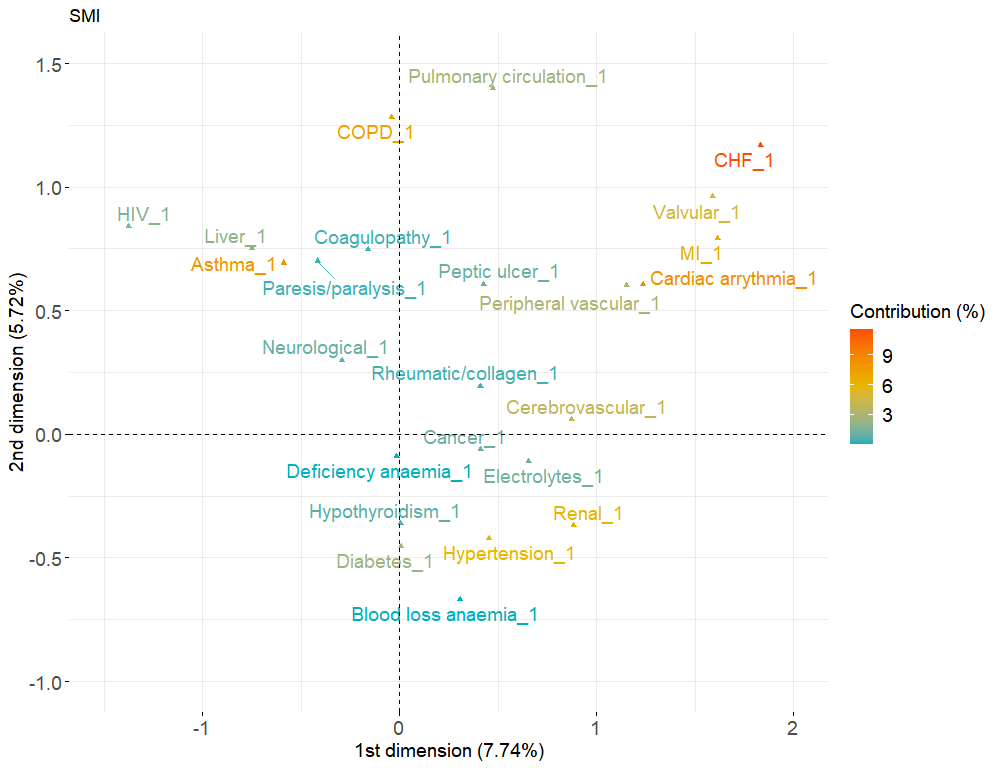


COPD: Chronic obstructive pulmonary disease, CHF: congestive heart failure, HIV: human immunodeficiency virus; MI: myocardial infarction; Electrolytes: Fluid and electrolyte disorders.
