# Supplementary material for: Clustering of physical health multimorbidity in people with severe mental illness: An accumulated prevalence analysis of United Kingdom primary care data
Source: PLoS Med. 2022 Apr 20;19(4):e1003976. doi: 10.1371/journal.pmed.1003976 (PMC9067697; doi:10.1371/journal.pmed.1003976)
Supplement: S1 Table — OR, odds ratio. (DOCX) [file pmed.1003976.s004.docx]

#### Supplement 4 Table: Odds ratios of physical health conditions in patients with ethnicity coded as missing, compared to those with ethnicity coded as White

| **Condition** | **Unadjusted OR (95%CI)** | **Demographic adjusted OR (95%CI)** | **Demographic and risk adjusted OR (95%CI)** |
| --- | --- | --- | --- |
| Asthma | 0.73 (0.72-0.75) p<0.001 | 0.72 (0.70-0.73) p<0.001 | 0.83 (0.81-0.84) p<0.001 |
| COPD | 0.58 (0.56-0.60) p<0.001 | 0.56 (0.54-0.58) p<0.001 | 0.72 (0.69-0.75) p<0.001 |
| Cardiac arrythmia | 0.74 (0.71-0.76) p<0.001 | 0.69 (0.66-0.71) p<0.001 | 0.79 (0.77-0.82) p<0.001 |
| Congestive heart failure | 0.89 (0.86-0.93) p<0.001 | 0.85 (0.81-0.89) p<0.001 | 1.03 (0.98-1.08) p=0.33 |
| Myocardial infarction | 0.86 (0.82-0.90) p<0.001 | 0.83 (0.78-0.87) p<0.001 | 1.02 (0.97-1.08) p=0.36 |
| Cerebrovascular disease | 0.90 (0.87-0.93) p<0.001 | 0.86 (0.83-0.89) p<0.001 | 0.97 (0.93-1.00) p=0.07 |
| Neurological disease | 0.78 (0.75-0.80) p<0.001 | 0.81 (0.78-0.84) p<0.001 | 0.85 (0.82-0.88) p<0.001 |
| Cancer | 0.82 (0.80-0.84) p<0.001 | 0.78 (0.76-0.80) p<0.001 | 0.86 (0.84-0.88) p<0.001 |
| Diabetes | 0.55 (0.54-0.57) p<0.001 | 0.54 (0.52-0.55) p<0.001 | 0.73 (0.71-0.74) p<0.001 |
| Hypothyroidism | 0.67 (0.65-0.70) p<0.001 | 0.68 (0.66-0.70) p<0.001 | 0.78 (0.76-0.81) p<0.001 |
| Liver disease | 0.68 (0.64-0.72) p<0.001 | 0.70 (0.66-0.75) p<0.001 | 0.88 (0.83-0.93) p<0.001 |
| Renal disease | 0.64 (0.62-0.65) p<0.001 | 0.53 (0.51-0.55) p<0.001 | 0.66 (0.64-0.69) p<0.001 |
| Peptic ulcer | 0.88 (0.84-0.92) p<0.001 | 0.87 (0.83-0.92) p<0.001 | 0.99 (0.94-1.04) p=0.60 |
| Rheumatic/collagen diseases | 0.79 (0.76-0.83) p<0.001 | 0.77 (0.74-0.81) p<0.001 | 0.87 (0.83-0.91) p<0.001 |
| Paresis/paralysis | 0.90 (0.82-0.99) p=0.03 | 0.91 (0.82-1.00) p=0.06 | 0.92 (0.83-1.02) p=0.12 |
| HIV | 0.49 (0.45-0.55) p<0.001 | 0.53 (0.48-0.59) p<0.001 | 0.60 (0.54-0.66) p<0.001 |
| Hypertension | 0.66 (0.65-0.67) p<0.001 | 0.57 (0.56-0.58) p<0.001 | 0.78 (0.76-0.79) p<0.001 |
| Peripheral vascular disease | 0.84 (0.80-0.88) p<0.001 | 0.81 (0.77-0.85) p<0.001 | 0.99 (0.93-1.04) p=0.60 |
| Pulmonary circulation disorders | 0.73 (0.69-0.78) p<0.001 | 0.73 (0.68-0.78) p<0.001 | 0.88 (0.82-0.95) p<0.001 |
| Valvular disease | 0.71 (0.67-0.75) p<0.001 | 0.66 (0.62-0.70) p<0.001 | 0.74 (0.70-0.78) p<0.001 |
| Deficiency anaemia | 0.72 (0.70-0.74) p<0.001 | 0.75 (0.72-0.78) p<0.001 | 0.82 (0.79-0.85) p<0.001 |
| Blood loss anaemia | 0.63 (0.46-0.86) p=0.003 | 0.73 (0.53-1.00) p=0.053 | 0.81 (0.59-1.12) p=0.20 |
| Coagulopathy | 0.72 (0.66-0.78) p<0.001 | 0.74 (0.67-0.81) p<0.001 | 0.84 (0.77-0.93) p<0.001 |
| Fluid and electrolyte disorders | 0.65 (0.62-0.69) p<0.001 | 0.64 (0.61-0.68) p<0.001 | 0.75 (0.71-0.79) p<0.001 |

CI: Confidence interval, COPD: Chronic obstructive pulmonary disease, HIV: Human immunodeficiency virus, OR: Odds rati0
