# Supplementary material for: Clustering of physical health multimorbidity in people with severe mental illness: An accumulated prevalence analysis of United Kingdom primary care data
Source: PLoS Med. 2022 Apr 20;19(4):e1003976. doi: 10.1371/journal.pmed.1003976 (PMC9067697; doi:10.1371/journal.pmed.1003976)
Supplement: S2 Table — aOR, adjusted odds ratio. (DOCX) [file pmed.1003976.s005.docx]

#### Supplement 5 table: Adjusted odds ratios of physical health conditions after multiple imputation for missing ethnicity

|  | **Demographic adjusted Odds Ratio (95% CI)** | | | **Demographic and risk adjusted Odds Ratio (95% CI)** | | |
| --- | --- | --- | --- | --- | --- | --- |
|  | **Schizophrenia** | **Bipolar** | **Other** | **Schizophrenia** | **Bipolar** | **Other** |
| Asthma | 1.08 (1.03-1.13) p<0.001 | 1.42 (1.37-1.47) p<0.001 | 1.24 (1.20-1.28) p<0.001 | 0.88 (0.84-0.92) p<0.001 | 1.16 (1.12-1.20) p<0.001 | 1.06 (1.03-1.10) p<0.001 |
| COPD | 1.83 (1.70-1.98) p<0.001 | 1.86 (1.75-1.97) p<0.001 | 1.35 (1.28-1.43) p<0.001 | 1.28 (1.18-1.39) p<0.001 | 1.35 (1.27-1.44) p<0.001 | 1.08 (1.02-1.15) p=0.009 |
| Cardiac arrythmia | 0.91 (0.83-1.00) p=0.04 | 1.11 (1.04-1.18) p=0.002 | 1.09 (1.03-1.15) p=0.002 | 0.86 (0.78-0.94) p=0.001 | 0.99 (0.93-1.05) p=0.70 | 1.05 (1.00-1.11) p=0.05 |
| Congestive heart failure | 1.05 (0.93-1.19) p=0.41 | 1.28 (1.17-1.40) p<0.001 | 1.10 (1.02-1.18) p=0.011 | 0.97 (0.86-1.10) p=0.67 | 1.12 (1.02-1.22) p=0.02 | 1.07 (0.99-1.15) p=0.07 |
| Myocardial infarction | 0.95 (0.84-1.09) p=0.48 | 1.03 (0.93-1.14) p=0.57 | 1.14 (1.06-1.23) p<0.001 | 0.86 (0.75-0.98) p=0.02 | 0.89 (0.81-0.99) p=0.03 | 1.11 (1.03-1.20) p=0.006 |
| Cerebrovascular disease | 1.27 (1.16-1.39) p<0.001 | 1.69 (1.59-1.80) p<0.001 | 1.48 (1.40-1.56) p<0.001 | 1.17 (1.07-1.28) p<0.001 | 1.52 (1.43-1.62) p<0.001 | 1.41 (1.34-1.49) p<0.001 |
| Neurological disease | 2.81 (2.63-3.01) p<0.001 | 2.59 (2.46-2.73) p<0.001 | 3.22 (3.08-3.37) p<0.001 | 2.55 (2.38-2.73) p<0.001 | 2.33 (2.20-2.46) p<0.001 | 2.93 (2.79-3.07) p<0.001 |
| Cancer | 0.81 (0.75-0.87) p<0.001 | 1.09 (1.03-1.14) p=0.001 | 0.85 (0.81-0.89) p<0.001 | 0.78 (0.72-0.84) p<0.001 | 1.02 (0.97-1.07) p=0.49 | 0.84 (0.80-0.88) p<0.001 |
| Diabetes | 2.14 (2.05-2.24) p<0.001 | 2.08 (2.01-2.16) p<0.001 | 1.52 (1.47-1.58) p<0.001 | 1.66 (1.58-1.74) p<0.001 | 1.52 (1.47-1.58) p<0.001 | 1.35 (1.30-1.40) p<0.001 |
| Hypothyroidism | 1.52 (1.41-1.65) p<0.001 | 2.56 (2.44-2.68) p<0.001 | 1.36 (1.29-1.43) p<0.001 | 1.41 (1.30-1.52) p<0.001 | 2.27 (2.16-2.38) p<0.001 | 1.33 (1.26-1.40) p<0.001 |
| Liver disease | 2.00 (1.81-2.21) p<0.001 | 1.75 (1.60-1.91) p<0.001 | 2.10 (1.95-2.26) p<0.001 | 1.10 (0.99-1.22) p=0.09 | 0.99 (0.90-1.08) p=0.80 | 1.12 (1.04-1.22) p=0.004 |
| Renal disease | 1.25 (1.16-1.35) p<0.001 | 1.63 (1.54-1.71) p<0.001 | 1.08 (1.03-1.13) p=0.002 | 1.18 (1.09-1.27) p<0.001 | 1.42 (1.34-1.49) p<0.001 | 1.07 (1.02-1.13) p=0.005 |
| Peptic ulcer | 0.90 (0.79-1.02) p=0.10 | 1.32 (1.21-1.44) p<0.001 | 1.33 (1.23-1.43) p<0.001 | 0.74 (0.65-0.84) p<0.001 | 1.10 (1.01-1.21) p=0.03 | 1.16 (1.07-1.25) p<0.001 |
| Rheumatic/collagen diseases | 0.71 (0.62-0.81) p<0.001 | 1.14 (1.05-1.24) p=0.001 | 0.98 (0.91-1.05) p=0.52 | 0.67 (0.59-0.77) p<0.001 | 1.05 (0.97-1.14) p=0.24 | 0.96 (0.89-1.03) p=0.28 |
| Paresis/paralysis | 1.44 (1.16-1.78) p<0.001 | 2.03 (1.76-2.35) p<0.001 | 2.16 (1.90-2.45) p<0.001 | 1.40 (1.13-1.74) p=0.002 | 1.95 (1.68-2.26) p<0.001 | 2.13 (1.86-2.43) p<0.001 |
| HIV | 1.15 (0.95-1.39) p=0.16 | 1.84 (1.62-2.08) p<0.001 | 1.81 (1.61-2.04) p<0.001 | 0.85 (0.70-1.03) p=0.09 | 1.39 (1.22-1.59) p<0.001 | 1.35 (1.19-1.53) p<0.001 |
| Hypertension | 0.88 (0.84-0.93) p<0.001 | 1.17 (1.12-1.21) p<0.001 | 0.91 (0.88-0.94) p<0.001 | 0.70 (0.66-0.74) p<0.001 | 0.85 (0.82-0.89) p<0.001 | 0.81 (0.78-0.84) p<0.001 |
| Peripheral vascular disease | 0.97 (0.85-1.12) p=0.72 | 1.28 (1.16-1.40) p<0.001 | 1.09 (1.01-1.19) p=0.03 | 0.80 (0.70-0.92) p=0.002 | 1.06 (0.97-1.17) p=0.20 | 1.00 (0.92-1.08) p=0.97 |
| Pulmonary circulation disorders | 1.41 (1.20-1.64) p<0.001 | 1.68 (1.51-1.88) p<0.001 | 1.45 (1.32-1.60) p<0.001 | 1.20 (1.02-1.40) p=0.03 | 1.37 (1.23-1.53) p<0.001 | 1.33 (1.20-1.47) p<0.001 |
| Valvular disease | 0.73 (0.62-0.87) p<0.001 | 1.02 (0.92-1.14) p=0.69 | 0.97 (0.89-1.06) p=0.50 | 0.71 (0.60-0.84) p<0.001 | 0.94 (0.85-1.05) p=0.28 | 0.96 (0.87-1.04) p=0.31 |
| Deficiency anaemia | 1.34 (1.25-1.44) p<0.001 | 1.41 (1.34-1.49) p<0.001 | 1.38 (1.31-1.44) p<0.001 | 1.27 (1.18-1.36) p<0.001 | 1.29 (1.22-1.36) p<0.001 | 1.33 (1.27-1.40) p<0.001 |
| Blood loss anaemia | 1.16 (0.63-2.14) p=0.64 | 0.91 (0.53-1.57) p=0.73 | 0.78 (0.48-1.28) p=0.33 | 1.08 (0.58-2.01) p=0.81 | 0.84 (0.48-1.46) p=0.54 | 0.77 (0.47-1.26) p=0.31 |
| Coagulopathy | 1.08 (0.88-1.32) p=0.48 | 1.47 (1.27-1.69) p<0.001 | 1.44 (1.27-1.63) p<0.001 | 0.91 (0.74-1.12) p=0.39 | 1.20 (1.04-1.39) p=0.01 | 1.25 (1.10-1.42) p<0.001 |
| Fluid and electrolyte disorders | 1.60 (1.43-1.79) p<0.001 | 2.29 (2.12-2.47) p<0.001 | 1.68 (1.57-1.80) p<0.001 | 1.39 (1.24-1.56) p<0.001 | 1.90 (1.76-2.05) p<0.001 | 1.54 (1.44-1.65) p<0.001 |

CI: Confidence interval, COPD: Chronic obstructive pulmonary disease, HIV: Human immunodeficiency virus, OR: Odds ratio
